# Kingdom-Wide Analysis of Fungal Small Secreted Proteins (SSPs) Reveals their Potential Role in Host Association

**DOI:** 10.3389/fpls.2016.00186

**Published:** 2016-02-19

**Authors:** Ki-Tae Kim, Jongbum Jeon, Jaeyoung Choi, Kyeongchae Cheong, Hyeunjeong Song, Gobong Choi, Seogchan Kang, Yong-Hwan Lee

**Affiliations:** ^1^Fungal Bioinformatics Laboratory, Seoul National UniversitySeoul, South Korea; ^2^Department of Agricultural Biotechnology, Seoul National UniversitySeoul, South Korea; ^3^Interdisciplinary Program in Agricultural Genomics, Seoul National UniversitySeoul, South Korea; ^4^Department of Plant Pathology and Environmental Microbiology, The Pennsylvania State UniversityUniversity Park, PA, USA; ^5^Center for Fungal Genetic Resources, Center for Fungal Pathogenesis, Plant Genomics and Breeding Institute, Research Institute of Agriculture and Life Sciences, Seoul National UniversitySeoul, South Korea

**Keywords:** fungi, lifestyle, secretome, small secreted proteins, effectors

## Abstract

Fungal secretome consists of various functional groups of proteins, many of which participate in nutrient acquisition, self-protection, or manipulation of the environment and neighboring organisms. The least characterized component of the secretome is small secreted proteins (SSPs). Some SSPs have been reported to function as effectors, but most remain to be characterized. The composition of major secretome components, such as carbohydrate-active enzymes, proteases, lipases, and oxidoreductases, appear to reflect the lifestyle and ecological niche of individual species. We hypothesize that many SSPs participate in manipulating plants as effectors. Obligate biotrophs likely encode more and diverse effector-like SSPs to suppress host defense compared to necrotrophs, which generally use cell wall degrading enzymes and phytotoxins to kill hosts. Because different secretome prediction workflows have been used in different studies, available secretome data are difficult to integrate for comprehensive comparative studies to test this hypothesis. In this study, SSPs encoded by 136 fungal species were identified from data archived in Fungal Secretome Database (FSD) via a refined secretome workflow. Subsequently, compositions of SSPs and other secretome components were compared in light of taxa and lifestyles. Those species that are intimately associated with host cells, such as biotrophs and symbionts, usually have higher proportion of species-specific SSPs (SSSPs) than hemibiotrophs and necrotrophs, but the latter groups displayed higher proportions of secreted enzymes. Results from our study established a foundation for functional studies on SSPs and will also help understand genomic changes potentially underpinning different fungal lifestyles.

## Introduction

Diverse groups of pathogenic fungi threaten plant health, whereas certain fungi, such as endophytes and mycorrhizal fungi, allow plants to explore new niches, manage biotic and abiotic stresses better, and/or efficiently acquire key nutrients. In both types of plant-fungus interactions, the outcome of interaction is influenced heavily by fungal secretomes, various proteins secreted or injected to plants (Girard et al., 2013). In the secretome, certain small secreted proteins (SSPs) are known to be responsible for disease development as virulence factors or cause resistance (*R*)-gene mediated defense as avirulence factors (Rep, 2005; Deller et al., 2011; Hacquard et al., 2012). Such SSPs are termed effector proteins and modulate key defense signaling pathways and downstream responses, to attenuate microbe-associated molecular pattern (MAMP) triggered immunity (MTI; Jones and Dangl, 2006). Plants have evolved to activate effector-triggered immunity (ETI) by sensing specific effectors or molecular changes caused by effectors, mainly using the nucleotide binding, leucine-rich repeat class of *R*-gene products (Dodds and Rathjen, 2010).

Initially, effectors were considered as virulence factors secreted by pathogens (van Esse et al., 2007; Stergiopoulos and de Wit, 2009; Lo Presti et al., 2015). However, it has become apparent that effector-mediated manipulation of MTI is required even for symbiotic associations, because microbial partners also display MAMPs (Zamioudis and Pieterse, 2012; Gourion et al., 2015). Many SSPs have been identified as putative effectors in beneficial plant-associated bacteria (Soto et al., 2006) and mutualistic fungi, such as *Glomus intraradices* (Kloppholz et al., 2011) and *Laccaria bicolor* (Plett et al., 2011), expanding the definition of effectors as secreted microbial products that facilitate the establishment of various plant-microbe associations ranging from beneficial to detrimental. Furthermore, SSPs that resemble effector proteins of pathogenic fungi have been identified in saprotrophic fungi, suggesting additional roles of SSPs (Rovenich et al., 2014; Seidl et al., 2015). Driven by the discovery of diverse putative effector proteins in fungi representing different lifestyles, several studies have analyzed the repertoires of putative secreted proteins encoded by various fungi and the potential relationship between their secretomes and lifestyles (Lowe and Howlett, 2012; Krijger et al., 2014; Meinken et al., 2014; Lo Presti et al., 2015). Analysis of the size of secretome relative to the total proteome in 48 fungal species by Lowe and Howlett (2012) suggested its potential relationship with lifestyles. Another comparative study by Meinken et al. (2014) proposed that the secretome prediction of previous study may be overestimated because only SignalP was used for the prediction, but they drew the same conclusion. However, these studies did not consider individual components of the secretome. The study by Krijger et al. (2014) suggested that phylogenetic position strongly influenced both the secretome size and its composition by analyzing 33 fungal species but did not include major secreted enzyme groups. In addition those displaying different modes of pathogenesis (biotroph, hemibiotroph, and necrotroph) were combined as a single lifestyle in the last two analyses. Lastly, the review on fungal effector proteins by Lo Presti et al. (2015) only considered plant cell wall degrading enzymes in order to mine putative effector proteins. The secretome contains not only effector proteins but also groups of enzymes involved in the breakdown of cell walls, self-protection or nutrient acquisition, such as carbohydrate-active enzymes (CAZymes), oxidoreductases, proteases, and lipases (Girard et al., 2013). Not surprisingly, biotrophs encode fewer CAZymes than hemibiotrophs and necrotrophs (Zhao et al., 2014). To investigate whether the composition and size of putative effectors correlates with different lifestyles, such enzymes should also be analyzed separately.

A wide range of validated and suspected protein effectors encoded by bacteria and oomycetes have been identified, which was facilitated by the conserved delivery machinery to plant cells (Cornelis and Van Gijsegem, 2000) and sequence motifs present in effectors (Whisson et al., 2007), respectively. Although, a conserved IGY motif has been identified in a novel SSP family of Dikarya fungi (Cheng et al., 2014), known fungal effectors do not show conserved features, hampering their identification (Rafiqi et al., 2012; Giraldo and Valent, 2013). One or more of the following features have been used to predict candidate effector proteins in fungal secretomes: (a) presence of the signal peptide, but no transmembrane domain or GPI-anchor sites; (b) small sized proteins (usually fewer than 300 amino acids) that are present only in specific species or isolates; (c) expression *in planta* or during infection; (d) rich in cysteine residues; and (e) presence of a conserved motif within effector candidates like in oomycete and fungal effectors (Birch et al., 2008; Godfrey et al., 2010; Zuccaro et al., 2011; Cheng et al., 2014). Using these features candidate effector proteins have been identified in three types of plant pathogenic fungi: biotrophs such as rusts (Duplessis et al., 2011), smuts (Schirawski et al., 2010), and *Blumeria graminis* (Spanu et al., 2010), hemibiotrophs including *Verticillium dahliae* (Santhanam and Thomma, 2013) and *Magnaporthe oryzae* (Kim et al., 2010), and necrotrophs including *Fusarium graminearum* (Brown et al., 2012) and *Sclerotinia sclerotiorum* (Guyon et al., 2014). However, direct comparisons between these fungi are hampered because different bioinformatics approaches and criteria have been used for the prediction of effector proteins. The lack of robust pipelines that can be applied to mine candidate effector proteins from rapidly increasing genome sequences of phylogenetically diverse fungi and limited *in planta* expression data for the genes encoding SSPs also have hampered large-scale comparative analyses of putative effectors.

In this study, we refined multiple secretome components with the focus on SSPs for 136 fungal species archived in Fungal Secretome Database (FSD; Choi et al., 2010) via a data extraction pipeline consisting of multiple programs. This refined data set was analyzed in the context of the phylogenetic position and lifestyle of individual species. Secreted enzymes that likely play important roles in colonizing host plants, including CAZymes, oxidoreductases, which are likely secreted for protection against host-produced reactive oxygen species (Chi et al., 2009), and lipases and proteases, which participate in nutrient acquisition and manipulation of host defense, were also compared. Resulting data helped determine which secretome components might function as major lifestyle determinants. In addition, we mined candidate effector proteins for functional validation and also showed the pattern of evolutionary changes associated with several known effector proteins.

## Materials and methods

### Phylogenetic analysis

The phylogenetic trees of 136 fungal species shown in Supplementary Figure S2 was constructed using CVTree v4.2.1 with k-tuple 7 (Xu and Hao, 2009). The tree only shows topology and ectopically positioned *Taphrina deformans* was manually curated based on NCBI Taxonomy. The lifestyle of each fungus was annotated based on literature review.

### Secretome data collection, refinement, and annotation

The SP, SP^3^ and SL classes of secretory proteins, which include proteins carrying a classical signal peptide, were downloaded from FSD (Choi et al., 2010). Hence, only the proteins secreted via canonical pathway were considered in this analysis. In order to predict SSPs, a two-step mining pipeline was employed. The first step, adopted from Brown et al. (2012), involves refining the secretome by selecting proteins predicted to be secreted by both ProtComp v9.0 (detected as secreted) and WoLF PSORT v0.2 (extr => 10; Horton et al., 2007), which are protein localization prediction programs trained with fungal data. Proteins that may be secreted but probably membrane bound were filtered out using Phobius v1.01 (TM = 1; SP = N; Käll et al., 2004), a program that detects signal peptides and transmembrane helixes, and UTProt (GPI-anchored = Y), a fungal specific GPI-anchor prediction tool (Cao et al., 2009). These programs were run on local Linux computers and the parameter settings were determined with various fungal effector proteins listed in the review by Stergiopoulos and de Wit (2009). The second step was grouping proteins within individual refined secretomes based on their predicted functions. To identify CAZymes, relevant HMM profiles from dbCAN release 3.0 (Yin et al., 2012) were employed. Oxidoreductases, lipases, and proteases were identified using BLASTP (*E*-value cutoff of 0.001) with individual refined secretomes as queries against BLAST databases of these enzyme sets. Fungal oxidoreductases were downloaded from Fungal Peroxidase Database (Choi et al., 2014). Sources for the lipase and protease datasets were the Lipase Engineering Database (Fischer and Pleiss, 2003) and MEROPS (Rawlings et al., 2014), respectively.

### Mining and annotation of SSPs

Protein length and cysteine content were analyzed using in-house Python scripts, and putative species-specific proteins were identified by running BLASTP against all other species, followed by BLASTP against NR database excluding itself to reduce false positives caused by the limited phylogenetic coverage of certain taxa. Both species-specific SSPs (SSSPs) and conserved SSPs (CSSPs) were annotated using pre-computed InterPro terms, which were retrieved from Comparative Fungal Genomics Platform 2.0 (CFGP 2.0; Choi et al., 2013), and mapped to PHI-base effector proteins using BLASTP (Urban et al., 2015).

### Clustering of CSSPs and molecular evolutionary analysis

All by all BLASTP analyses were performed with CSSPs and PHI-base effector proteins prior to Markov Cluster Algorithm (MCL) clustering. Resulting data were clustered via MCL with the inflation option 1.4 for high granularity (Enright et al., 2002), which produced the least number of singletons. The predicted protein families containing PHI-base effectors were analyzed further. Proteome data from CFGP 2.0 were used to construct species trees, and proteins in each of the analyzed families were used to construct gene trees. CVtree v4.2.1 was used for fungal species tree with k-tuple 7 (Xu and Hao, 2009), ClustalW in MEGA 6.06 was used for alignment of proteins, and maximum-likelihood gene trees were constructed using the default setting (Hall, 2013). After reconciling the species and gene trees using Notung 2.6 (Chen et al., 2000), potential gene duplication and loss events were annotated.

## Results

### Analyzed species cover diverse taxa and lifestyles

Genome sequences of the 136 fungal species used in this study (Supplementary Table S1) are publicly available. The taxa covered include Microsporidia, Zygomycota, Glomeromycota, Ascomycota, and Basidiomycota (Table 1). The lifestyles represented include animal pathogens, biotrophic, hemibiotrophic and necrotrophic plant pathogens, symbionts, and saprotrophs (Table 1). The necrotrophs were further divided into crop-infecting and wood-decaying types. The symbionts are fungi associated with plants and resulting beneficial/mutualistic effects in the interactions. These include both ecto- and arbuscular mycorrhizal fungi, endophytes, and a plant growth promoting fungus with symbiotic activity (Vargas et al., 2009).

**Table 1:** **Taxonomic distribution and lifestyle of the species analyzed in this study**.

**Phylum**	**Subphylum (Division/Class)**	**Number of species**	**lifestyle**	**Number of species**
Ascomycota	Pezizomycotina	72	Animal pathogen	27
	Saccharomycotina	10		
	Taphrinomycotina	4	Biotroph	10
Basidiomycota	Agaricomycotina	34	Hemibiotroph	9
	Pucciniomycotina	4	C-necrotroph	23
	Ustilaginomycotina	5	W-necrotroph	26
Glomeromycota	(Glomeromycetes)	1	Symbiont	6
Microspora	(Microsporidia)	5		
Zygomycota	Mucoromycotina	1	Saprotroph	35
Total		136		136

*C-necrotroph, Crop-infecting necrotroph; W-necrotroph, Wood-decaying necrotroph*.

### Refined fungal secretomes show high degree of size variance

**Figure 1:**
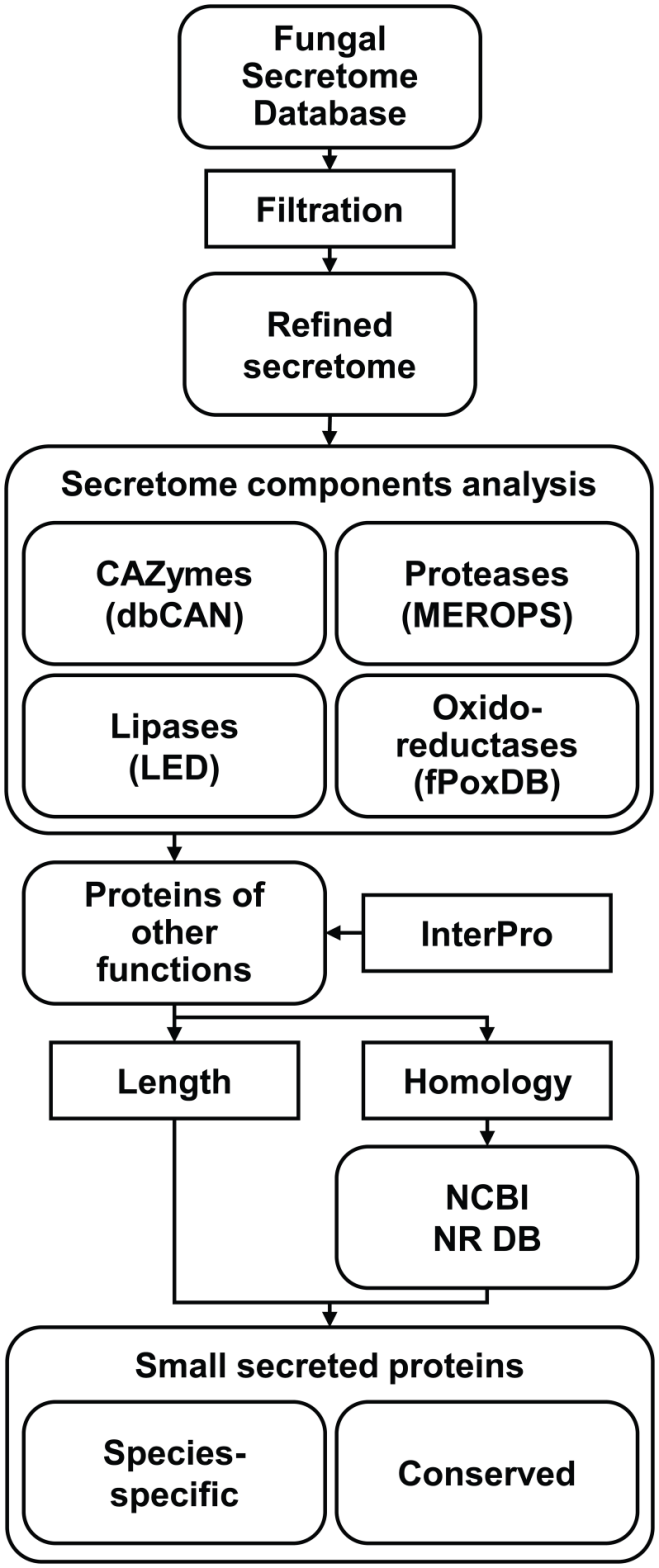
**Pipeline used to refine secretomes and mine small secreted proteins (SSPs)**. Chosen secretomes were downloaded from Fungal Secretome Database (FSD) and refined. The refined secretomes were then divided into four classes of enzymes, including CAZymes, proteases, lipases, and oxidoreductases, and proteins of undefined function. To predict functions of the latter group of proteins, InterPro analysis was performed, and the presence of signatures frequently associated with effectors, including short length and taxon-specific distribution, was also analyzed. To reduce false species-specific proteins due to the limited phylogenetic coverage of certain taxa, BLASTP against NCBI NR database was performed. SSPs were divided into species-specific SSPs (SSSPs) and conserved SSPs (CSSPs) (see Materials and Methods).

Since, fungal effectors are expected to be secreted into the host apoplastic space or cytoplasm, mining fungal secretomes is the first step for their identification. Secretome data for most sequenced fungi have already been archived in FSD (Choi et al., 2010), an online platform that was built to identify and archive secreted proteins via six different programs detecting extracellular proteins and a trans-membrane helix detection program. The predicted secretomes were then categorized into three different classes. To predict *bona fide* SSPs, the total secretome data from FSD were further refined via additional filtrations (Figure 1). Putatively membrane-bound proteins were eliminated using additional programs that detect transmembrane-helixes and GPI-anchors (see Materials and Methods).

After this refinement, the size of secretome was reduced by 33.7% on average compared to that predicted using only SignalP 3.0 and by 70.9% compared to the total secretome predicted by the pipeline used for building FSD (Supplementary Figure S1). The number of proteins in refined secretomes ranged from 19 (*Pneumocystis jirovecii*, an opportunistic human pathogen) to 1940 (*Auricularia subglabra*, a wood-decaying necrotroph; Supplementary Table S1). The refined secretome accounted for 5.5% of the total proteome on average, with the lowest being 0.5% (*P. jirovecii*) and the highest being 11.0% (*Magnaporthe oryzae*, a hemibiotrophic plant pathogen). Both *P. jirovecii* and *M. oryzae* belong to the phylum Ascomycota, illustrating high degrees of variance within individual phyla (Supplementary Figure S1).

### Patterns observed among refined secretomes in the context of phylogenetic positions and lifestyles

**Figure 2:**
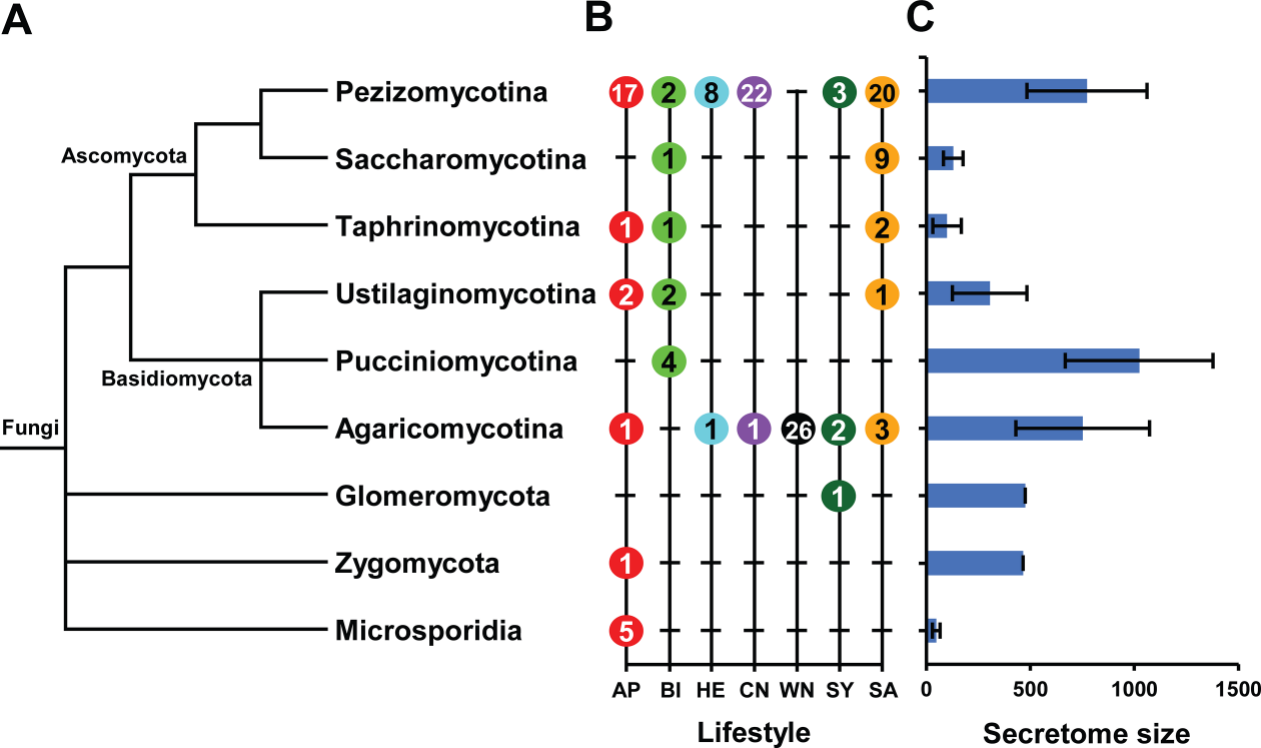
**The taxa and lifestyles represented and average size of refined secretomes for individual taxa. (A)** A condensed phylogenetic tree illustrates the taxonomic position and lifestyle of 136 fungal species. The full species tree is available in Supplementary Figure S2. **(B)** Number of species included and the lifestyles represented for each taxon are shown. AP, Animal pathogen; BI, Biotroph; HE, Hemibiotroph; CN, Crop-infecting necrotroph; WN, Wood-decaying necrotroph; SY, Symbiont; SA, Saprotroph. **(C)** Average sizes and size range of refined secretomes for individual taxa are noted.

Several general patterns associated with the size of refined secretome were observed (Figures 2A,B). On average, fungi belong to Pucciniomycotina encode the largest refined secretomes, whereas Microsporidia code for the smallest ones (Figure 2C). However, the size of refined secretome also varied widely within individual taxa as illustrated in Supplementary Figure S2. For example, in Ascomycota, the species belong to Pezizomycotina have much larger secretomes than those of Saccharomycotina and Taphrinomycotina, and in Basidiomycotia, the species belong to Pucciniomycotina have larger secretomes than those of Ustilaginomycotina.

**Figure 3:**
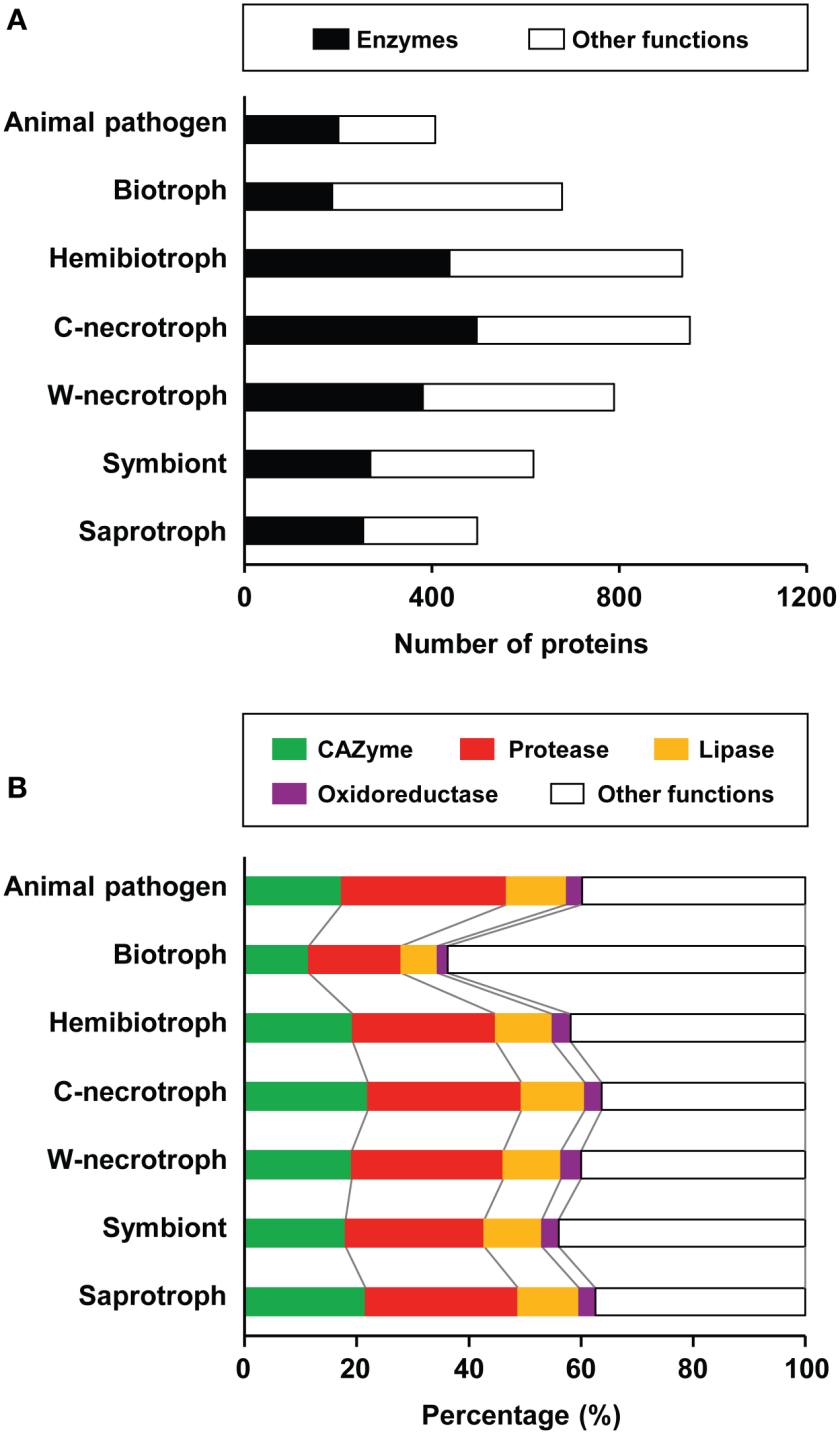
**Composition of refined secretomes in the context of different lifestyles. (A)** Average sizes of the refined secretome for each of the lifestyles represented is shown. The numbers of the refined secretome for individual species are in Supplementary Table S1. The enzymes correspond to CAZymes, proteases, lipases, and oxidoreductases. The proteins of other functions refer those that did not map to any of the four enzyme databases. C-necrotroph and W-necrotroph correspond to crop-infecting necrotroph and wood-decaying necrotroph, respectively. **(B)** Proportions of four enzyme classes and the proteins of other functions in the refined secretome are shown.

We analyzed the composition of refined secretomes among groups that represent different lifestyles to investigate their relationship. On average, the size of refined secretome for plant-associated species was larger than those of saprotrophs and animal pathogens (Figure 3A). Among the plant-associated species, pathogens encode larger refined secretomes than symbionts. Among the pathogens, crop-infecting necrotrophs code for the largest refined secretome, followed by hemibiotrophs, wood-decaying necrotrophs, and biotrophs. Proportions of CAZymes, proteases, lipases, and oxidoreductases in 136 species were analyzed in relation to their lifestyles (Figure 3B; Supplementary Table S1). Although these enzymes facilitate nutrient acquisition and defense against reactive oxygen species from host (Rogers et al., 1994; Sreedhar et al., 1999; Chi et al., 2009; Blümke et al., 2014), they were not considered for mining effector-like SSPs as in Rep (2005). On average, 46.6% of the refined secretome corresponded to these enzymes. Biotrophs display the lowest proportion for all four types of enzymes, and the highest proportion for all types, except oxidoreductases, was observed in crop-infecting necrotrophs. Wood-decaying necrotrophs exhibit the highest proportion of oxidoreductases. Animal pathogens have higher proportion of proteases than saprotrophs, symbionts, and plant pathogens except necrotrophs, suggesting the importance of proteases in animal pathogenesis and necrosis of plants.

### Patterns associated with effector-like SSPs

Most effector-like SSPs belong to the proteins of other functions. Accordingly, we first removed the four groups of enzymes from the refined secretomes before identifying SSPs (Figure 1). Subsequently, three features, including short length (≤300 aa), species-specific distribution pattern, and cysteine enrichment, were used to identify effector-like SSPs in the proteins of other functions.

**Figure 4:**
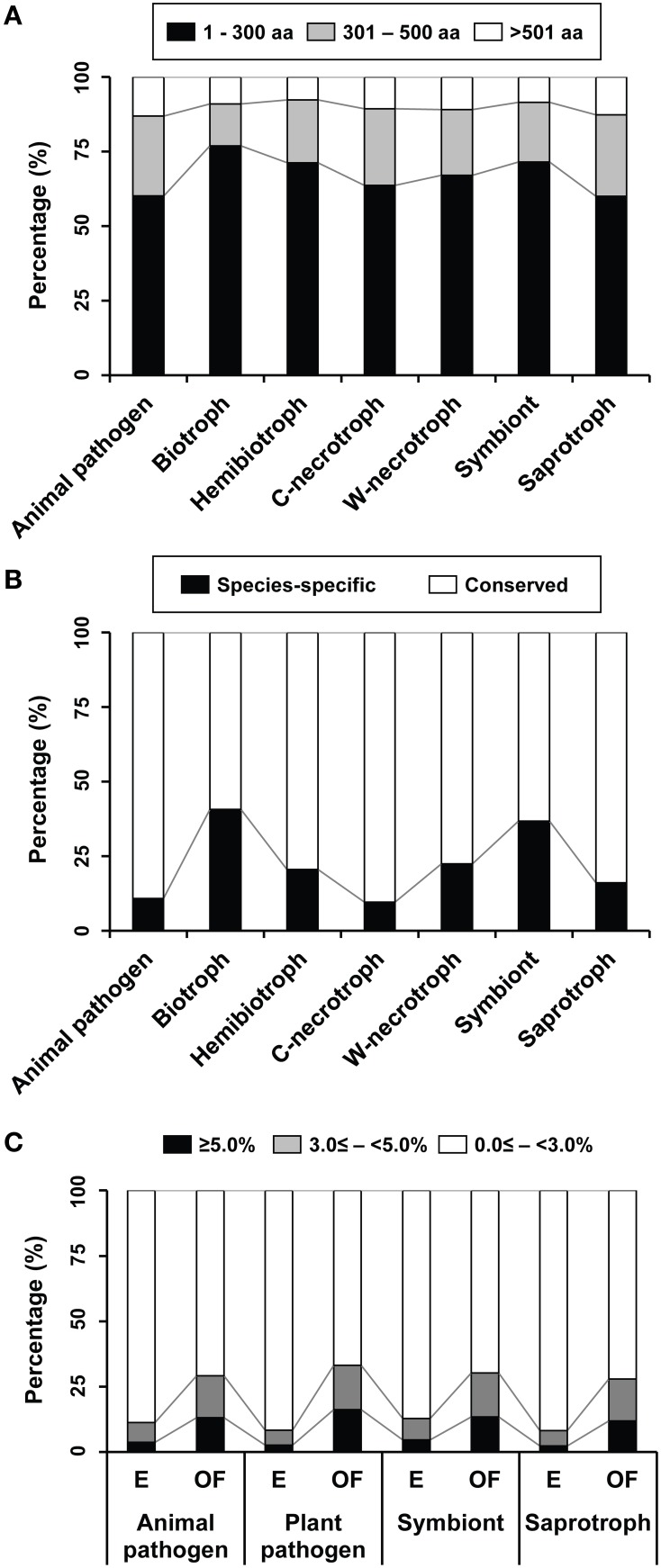
**Proportions of proteins of other functions that display characteristics typically associated with effectors in groups of species representing different lifestyles. (A)** Proportions of proteins in three size classes are shown. Those with 300 aa or less are considered as small secreted proteins (SSPs). C-necrotroph and W-necrotroph correspond to crop-infecting necrotroph and wood-decaying necrotroph, respectively. **(B)** Distribution patterns of species-specific and conserved proteins in individual lifestyles are shown. **(C)** Distribution of cysteine content in the four groups of enzymes (E) and the proteins of other functions (OF). Proteins with the cysteine content being equal or greater than 3% of the aa resides are considered as cysteine-rich.

Proteins shorter than 300 aa are abundant in all species (Figure 4A) with the exception of four saprophytic fungi, including *Schizosaccharomyces pombe, Pichia pastoris, Spathaspora passalidarum*, and *Ophiostoma piceae* (Supplementary Figure S3). In general, biotrophs have the most abundant SSPs. On other hands, proportions of species-specific proteins in the proteins of other functions varied between individual species (Supplementary Figure S4). In general, species that are intimately associated with living plant tissues, such as biotrophs and symbionts, have greater numbers of species-specific proteins than those with different lifestyles (Figure 4B). This suggests that species-specific presence of effectors probably arose via co-evolution with the hosts (Stergiopoulos et al., 2012). The species-specific proteins accounted for 25–50% of the proteins of other functions in 27 species and for over 50% in six species (Table 2). Many wood-decaying necrotrophs have large proportions (but not exceeding 50%) of species-specific secreted proteins. Proportions among symbionts typically ranged from 25 to 50%, but it is over 50% in *Rhizophagus irregularis*, which is the only Glomeromycota symbiont (Tisserant et al., 2012, 2013). Among the animal pathogens, three species belonging to Microsporidia (*Enterocytozoon bieneusi, Nosema ceranae*, and *Antonospora locustae*) and one Ascomycota species (*P. jirovecii*) displayed large proportions (>50%; Table 2).

**Table 2:** **List of species with high proportion of species-specific secreted proteins in the proteins of other functions**.

**Animal Pathogen**	**Biotroph**	**Hemibiotroph**	**C-necrotroph**	**W-necrotroph**	**Symbiont**	**Saprotroph**
**PROPORTION 25–50%**						
*E. bieneusi*	*P. graminis*	*L. maculans*	*R. solani*	*B. adusta*	*L. bicolor*	*C. cinerea*
*P. jirovecii*	*P. striiformis*	*M. oryzae*	*S. nodorum*	*B. botryosum*	*P. indica*	*B. compniacensis*
	*T. deformans*	*M. graminicola*		*Dacryopinax* sp.	*T. melanosporum*	*P. confluens*
				*G. marginata*		*Y. lipolytica*
				*H. irregulare*		
				*J. argilacea*		
				*P. carnosa*		
				*P. chrysosporium*		
				*S. lacrymans*		
				*T. mesenterica*		
**PROPORTION** >**50%**						
*A. locustae*	*B. graminis*				*R. irregularis*	
*N. ceranae*	*M. laricis-populina*					
	*M. osmundae*					

*C-necrotroph, Crop-infecting necrotroph; W-necrotroph, Wood-decaying necrotroph*.

The proteins of other functions and the four enzymes in the refined secretomes were classified into three groups based on their cysteine content, including 0.0 ≤ − <3.0%, 3.0 ≤ − <5.0%, and ≥5.0%, and average percentages of these groups in four lifestyles were compared (Figure 4C). Proteins with 3% or more cysteine were considered cysteine-rich in earlier studies (Stergiopoulos and de Wit, 2009; Saunders et al., 2012). However, a more stringent criterion, over 5%, was also used (Brown et al., 2012; Krijger et al., 2014). The general trend was that both classes of cysteine-rich proteins were more abundant among the proteins of other functions than the enzymes regardless of any lifestyles.

Effector-like SSPs were divided into two classes. Proteins of 300 aa or shorter that appeared to be encoded only by one species were considered species-specific SSPs (SSSPs); a total of 8275 SSSPs were found from 133 species. The fasta formatted SSSP sequences available in Supplementary Data Sheet 1. Three species, including two animal pathogens (*Malassezia sympodialis* and *Nematocida parisii*) and a saprotroph (*Kluyveromyces lactis*), did not have any SSSPs. The remaining SSPs were encoded by at least two species and were termed conserved SSPs (CSSPs), which may correspond to general fungal proteins or elicitors.

### Functional annotation of SSPs using interpro terms and the known effector proteins rarely reveals their functions

InterPro domain analysis was performed to predict potential functions of both SSSPs and CSSPs. Most SSPs, 7960 out of 8275 (96.2%), displayed no defined InterPro terms with only 315 SSPs being annotated with 97 different terms (Supplementary Table S2). Among the annotated SSSPs, the most commonly found InterPro term is membrane insertase YidC (IPR019998), which was found in 145 proteins. Other terms that are potentially related to pathogenicity and were found at least twice include IPR008427 (extracellular membrane protein, CFEM domain), IPR003172 (MD-2-related lipid-recognition domain), and IPR016191 (ribonuclease/ribotoxin).

Since the InterPro terms did not suggest any specific functions in association with pathogenicity, 49 known effector proteins from 11 fungi were retrieved from the PHI-base (Urban et al., 2015) and mapped to SSSPs (Supplementary Table S3). Nine effector proteins were mapped to four species with a bit score >50 and *e*-value < 1.e-3 (Table 3). Except MGG_10556T0 with a C2H2-type zinc finger domain, which resembles *M. oryzae* avirulence factor AVR-Pii, the others contained no known domains.

**Table 3:** **Species-specific effector proteins found among SSSPs**.

**PHI-base accession**	**Effector name**	**Species**	**Locus ID^#^**	**Score (Bits)**	***E*-value**	**InterPro domain**
PHI:2136	AVR-Pii	*M. oryzae*	MGG_10556T0	54.3	5.00E-12	IPR007087 IPR015880
PHI:2137	AVR-Pik	*M. oryzae*	MGG_15972T0	229	4.00E-79	–
PHI:2426	Avr2	*F. oxysporum*	FOXG_16398T0	333	7.00E-119	–
PHI:2898	BEC1011	*B. graminis*	BGHDH14_bgh02874	127	3.00E-39	–
PHI:2899	BEC1038	*B. graminis*	BGHDH14_bgh04220	338	7.00E-121	–
PHI:2900	BEC1016	*B. graminis*	BGHDH14_bgh06518	235	8.00E-82	–
PHI:2902	BEC1018	*B. graminis*	BGHDH14_bgh03694	239	2.00E-83	–
PHI:2903	BEC1054	*B. graminis*	BGHDH14_bgh02874	245	3.00E-85	–
PHI:2919	AvrLm11	*L. maculans*	Lema_T119060.1	203	1.00E-69	–

# *Locus ID of SSSPs matched to effectors from PHI-base by BLASTP*.

*IPR007087: Zinc finger, C2H2-type, IPR015880: Zinc finger, C2H2-like*.

CSSPs were clustered with 49 PHI-base effectors via MCL clustering analysis, and 2786 families containing 19,342 proteins were identified. Among them, 13 families contained at least one PHI-base effector (Table 4). Of these families, only one is associated with InterPro term related to fungal pathogenicity. For example, proteins in the family containing *M. oryzae* effector MgSM1 carry a well-known domain (cerato-platanin, IPR010829; Chen et al., 2013). In total, 22 out of 49 PHI-base effectors were either matched to a single protein or clustered within a protein family (Supplementary Table S3). The small number of matches may be due to the strain-specific presence of many effectors.

**Table 4:** **Protein families that contain known effector proteins**.

**PHI-base accession (Effector name)**	**Species^#^**	**Number of proteins**	**Number of taxa**	**InterPro domain**
PHI:2118 (MgSM1)	*M. oryzae*	201	91	IPR009009 IPR010829
PHI:2901 (BEC1040)	*B. graminis*	97	51	–
PHI:2897 (BEC1019)	*B. graminis*	34	33	–
PHI:2896 (BEC1005)	*B. graminis*	12	9	IPR017853
PHI:325 (ACE1)	*M. oryzae*	9	9	IPR001509 IPR016040
PHI:71 (ECP2)	*C. fulvum*	7	4	–
PHI:2331 (Ave1)	*V. dahliae*	5	4	–
PHI:1132(AvrLm4-7)	*L. maculans*	5	2	–
PHI:2696 (NIP2)	*R. commune*	4	3	–
PHI:2281 (avrLm1)	*L. maculans*	3	2	–
PHI:1131 (AvrLm6)	*L. maculans*	2	2	–
PHI:379 (SIX1)	*F. oxysporum*	2	2	–
PHI:2744 (Pit2)	*U. maydis*	2	2	–

# *Species which the PHI-base effectors originated. IPR009009: Barwin-related endoglucanase, IPR010829: Cerato-platanin, IPR017853: Glycoside hydrolase, superfamily, IPR001509: NAD-dependent epimerase/dehydratase and IPR016040: NAD(P)-binding domain*.

### Genomic contexts of SSSP-coding genes

The genomic regions containing the known host-specific virulence genes have been shown to have sparsely distributed genes and AT-rich (Schmidt and Panstruga, 2011). We examined the genomic contexts of SSSP-coding genes in 59 species with number of contigs less than 500 (Supplementary Table S4). The average number of genes and the mean AT-content in each of the 100 kb segments of their genomes were calculated as references. The corresponding data for each the 100 kb windows containing SSSP-coding gene(s) were compared with the reference data.

The gene density around SSSP-coding genes was too variable to establish a clear trend in the context of lifestyles. However, many SSSP-coding genes in a mycorrhizal symbiont *L. bicolor*, a plant growth promoting fungus *Trichoderma virens* and most wood-decaying necrotrophs were often located in regions with low gene density (Supplementary Table S4). Overall, the SSSP-coding genes did not appear to be concentrated within specific genomic region(s). On the other hand, the AT-content around most SSSP-coding genes was clearly lower than the total AT-content of the genomes of these species, except that two animal pathogens (*Cryptococcus neoformans* and *P. jirovecii*), one biotroph (*Mixia osmundae*), and one saprotroph (*Wallemia sebi*) had higher AT-contents at 52, 72, 44, and 60%, respectively (Supplementary Table S4).

### The number of SSSPs correlates with the proteome size, lifestyle, and taxonomic position

**Figure 5:**
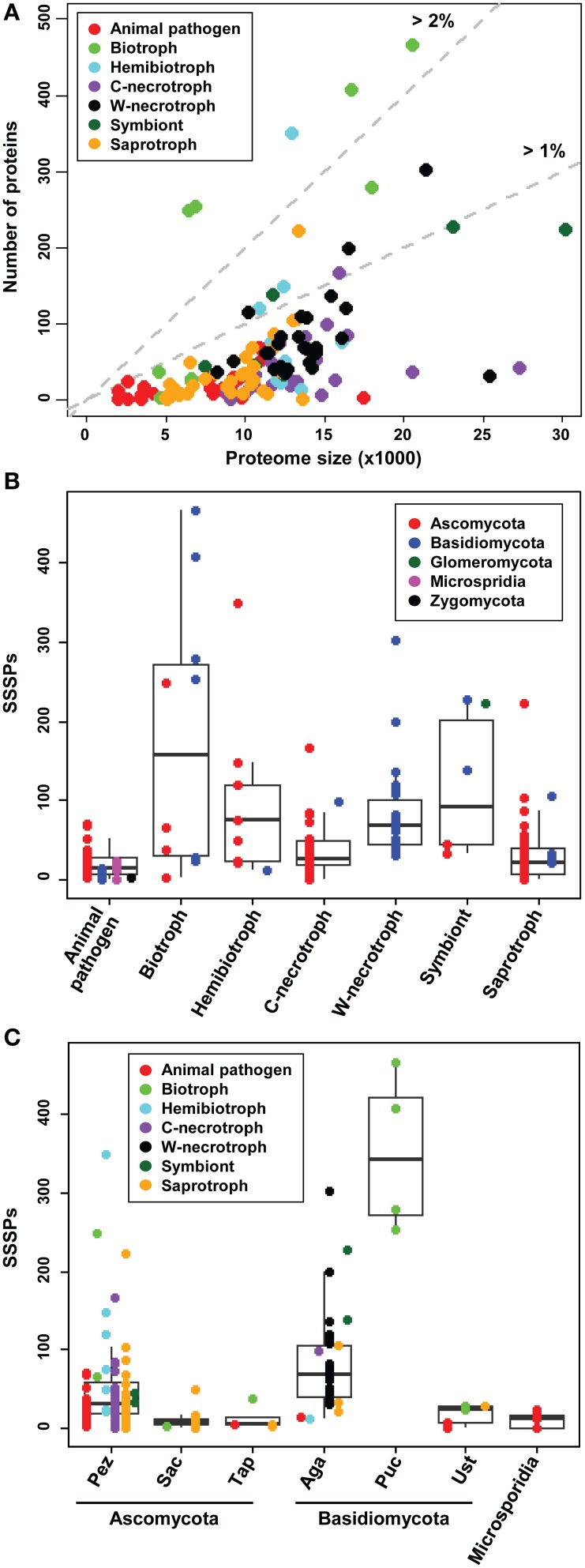
**Number of SSSPs in relation to the lifestyles and taxonomic positions of the analyzed species. (A)** The number of SSSPs and the size of predicted total proteome for individual species are shown. **(B)** The number of SSSPs among species representing different lifestyles. **(C)** The number of SSSPs encoded by the species in different taxa. The subphyla Glomeromycota and Zygomycota, represented by only one species each, are not included. Pez, Pezizomycotina; Sac, Saccharomycotina; Tap, Taphrinomycotina; Aga, Agaricomycotina; Puc, Pucciniomycotina; Ust, Ustilaginomycotina. C-necrotroph and W-necrotroph correspond to Crop-infecting necrotroph and Wood-decaying necrotroph, respectively.

Several studies have reported that the size of fungal secretome correlates with lifestyle (Lowe and Howlett, 2012; Meinken et al., 2014; Lo Presti et al., 2015) and that even stronger correlation exists with phylogenetic position (Krijger et al., 2014). The former studies reported that animal pathogens and saprotrophs have similarly-sized secretomes, but their secretomes are smaller than those in plant pathogens. We assessed the size of SSSPs in individual species to determine whether similar patterns exist. The relationships between the predicted proteomes and refined secretome components are shown in Supplementary Figure S5. The pattern was similar to that observed in a previous study (Lowe and Howlett, 2012). However, the relationship between SSSPs and the total proteome was markedly different (Figure 5A). Biotrophs, symbionts, and some hemibiotrophs usually have larger numbers of SSSPs than animal pathogens, saprotrophs, and necrotrophs. The larger numbers of SSSPs in the former group, which are intimately associated with plants, support the hypothesis that these proteins are important for manipulating plant hosts.

The number of SSSPs within species ranged from 0 to 466. Although not all plant-associated fungi have higher numbers of SSSPs than saprotrophs and animal pathogens (Figure 5B), in general, animal pathogens and saprotrophs had fewer SSSPs compared to plant pathogens. Within plant pathogens, numbers of SSSPs encoded by crop-infecting necrotrophs are similar to those encoded by saprotrophs, and biotrophs generally have larger numbers than these groups. Within biotrophs, four Pucciniomycotina species (*Puccinia graminis, P. striiformis, Melampsora laricis-populina*, and *M. osmundae*) and *B. graminis* (Ascomycota) have the highest numbers. In contrast, two species in Ustilagomycotina (*Ustilago maydis* and *Sporisorium reilianum*) and three Ascomycota species (*Cladosporium fulvum, T. deformans*, and *Ashbya gossypii*) encode small numbers of SSSPs, similar to those in saprotrophs. In addition, wood-decaying necrotrophs and hemibiotrophs have similar numbers of SSSPs.

The range of SSSPs numbers was also analyzed with regard to taxonomic positions (Figure 5C). The mean number within Basidiomycota is 100, which is higher than the mean in Ascomycota (43). Within Basidiomycota, Pucciniomycotina encodes the most SSSPs. Within Ustilaginomycotina, two biotrophs (*U. maydis* and *S. reilianum*) contained greater numbers of SSSPs compared to other members. In Ascomycota, the range was widest in Pezizomycotina with many outliers being present at both sides of the mean. Three hemibiotrophs, including *M. oryzae, Leptosphaeria maculans*, and *Mycosphaerella graminicola*, one nectrotroph *Stagonospora nodorum*, one biotroph *B. graminis*, and one saprotroph *Pyronema confluens* encode much larger SSSPs than the mean. Saccharomycotina and Taphrinomycotina encode noticeably smaller SSSPs than Pezizomycotina.

The biotrophs in Pezizomycotina code for relatively large numbers of SSSPs. Similarly, *T. deformans*, the only biotroph in Taphrinomycotina, also shows a high number of SSSPs. However, *A. gossypii*, the only biotroph in Saccharomycotina, has a lower number and proportion of SSSPs compared to other members (Figure 5C).

### Evolution of known effector proteins belonging to CSSP families suggests their other roles

**Figure 6:**
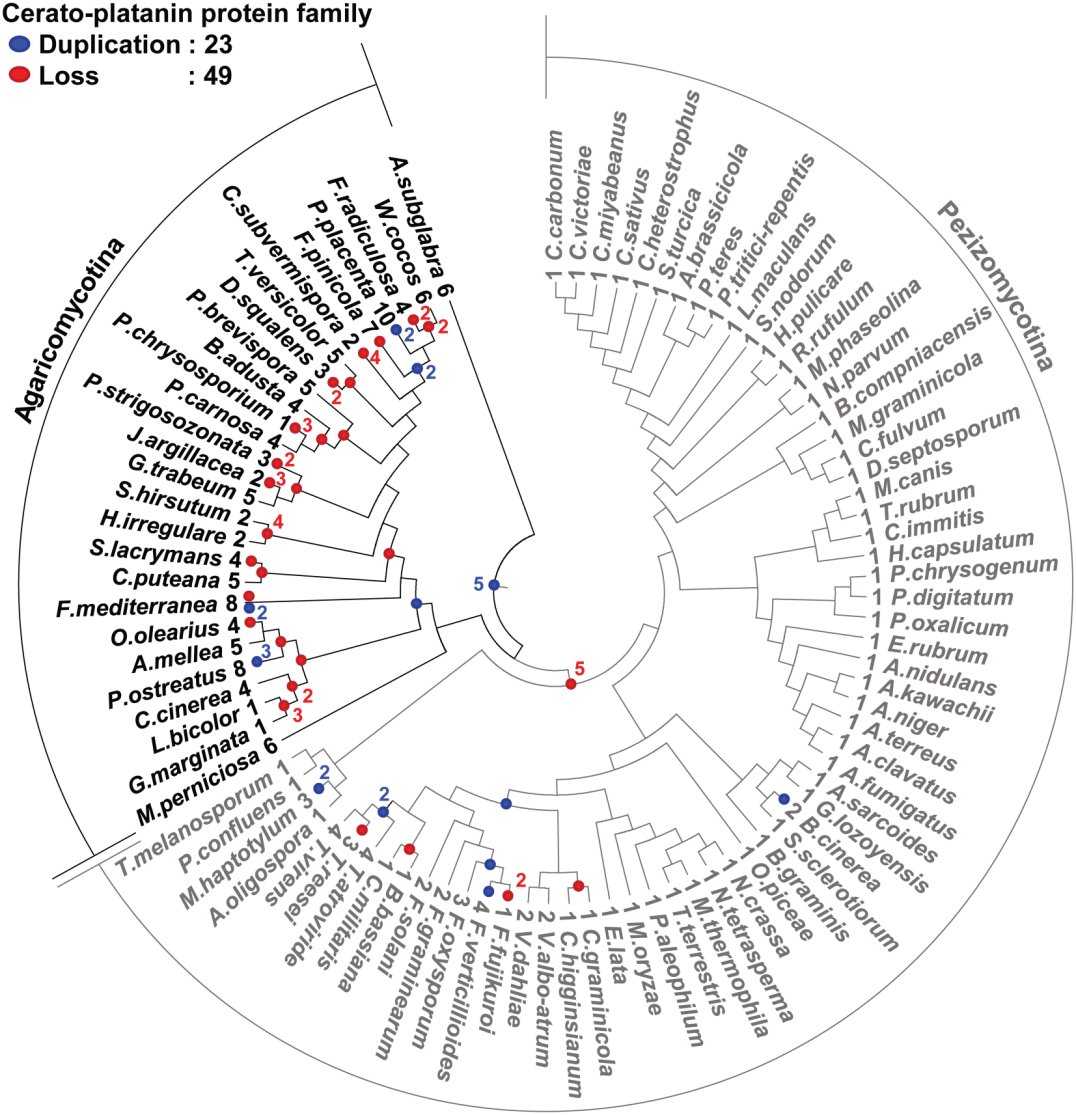
**Evolution of the protein family carrying the cerato-platanin domain**. The protein family with this domain is the largest cluster identified by MCL analysis with MgSM1 effector protein. Members of this family are found in 91 species within Agaricomycotina and Pezizomycotina. The cerato-platanin protein tree was reconciled with the species tree using the Notung software. The number of genes present in each species is shown at the end of nodes before species name. Gene gains (23 events) and losses (49 events) are noted by blue and red dots, respectively.

We predicted 13 families of CSSPs containing PHI-base effectors (Table 4). Among them, families containing *M. oryzae* effector MgSM1 and the *B. graminis* effectors were found in the most taxa, and the evolution of these families of CSSPs was analyzed. Genes encoding proteins with a cerato-platanin domain (IPR009009) were conserved in 91 species, all belonging to Pezizomycotina or Agricomycotina (Figure 6). In total, 23 gene duplications and 49 gene losses were observed in the family, but the gene duplications and losses were skewed toward Agaricomycotina, which consisted mostly of wood-decaying necrotrophs. However, these genes were not found in Ustilaginomycotina and Pucciniomycotina, the other subphyla of Basidiomycota that mostly consisted of biotrophic species. The genes encoding proteins that carry the ancestral cerato-platanin domain have undergone at least five duplication events, but many of the genes also have been lost in multiple lineages. As a consequence, 33 species in Dothideomycetes and Eurotiomycetidae contained only one such gene. However, copies in the necrotrophic Sordariomycetes, *Botrytis cinerea* and *Fusarium* spp. have undergone duplication events. The wide distribution of the genes encoding cerato-platanin domain proteins in species with pathogenic lifestyles suggested that their products play an important role in pathogenesis. However, numbers of this group were also found in 4 symbionts, 2 nematophagous fungi, and 19 saprotrophs, suggesting other roles associated with them. The gene family identified using the *B. graminis* effector candidate BEC1040 has gone through 16 duplications and 12 losses, and its numbers were present in 50 species in Ascomycota and *Punctularia strigosozonata* in Basidiomycota (Supplementary Figure S6A). Only one duplication event occurred in the family identified with BEC1019 (Supplementary Figure S6B), but both duplication and loss events occurred in the family identified with BEC1005 (Supplementary Figure S6C). Overall, many CSSPs that resemble known effectors were identified in non-plant pathogens, raising the possibility that they are remnants of degenerated genes or play roles other than facilitating plant infection.

## Discussion

Rapid progresses in sequencing fungal genomes, in combination with various “omics” tools, have facilitated large-scale comparative genomic analyses to uncover the genetic and evolutionary basis of various traits or functions of fundamental and practical significance. In this study, we developed a pipeline for mining SSPs as effector candidates from fungi with different lifestyles and taxonomic positions in order to conduct their kingdom-wide comparative analysis. It has been commonly hypothesized that biotrophs and symbionts secrete more effectors than necrotrophs, as biotrophic associations require the modulation of the host defense system to keep host cells alive for nutrient acquisition while preventing the launch of strong defense responses. Since necrotrophs utilize CAZymes and toxins to kill host cells to obtain nutrient, such manipulations of host defense likely play less critical roles. Because only a small number of fungal proteins in selected plant pathogens have been identified as effectors, we tested this hypothesis by comparing SSSPs as effector candidates.

We refined secretomes to identify SSSPs as previously reported in analyzing the secretome of *F. graminearum* (Brown et al., 2012). In addition, we mined and compared other components within the refined secretomes to investigate any lifestyle-associated genomic adaptations. Three previous studies examined potential relationships between the secretome and lifestyle (Lowe and Howlett, 2012; Krijger et al., 2014; Lo Presti et al., 2015). However, Lowe and Howlett (2012) and Lo Presti et al. (2015) used only one signal peptide prediction program for mining secretomes and Krijger et al. (2014) did not eliminate putative membrane-bound proteins, which likely inflated the size of secretomes. Our refined secretome pipeline more rigorously identified secretory proteins via two additional protein localization detection programs and the elimination of transmembrane and GPI-anchor proteins. For comparison, the number of SSSPs and the effector candidates in powdery mildew predicted by Spanu et al. (2010) is the same, and the refined secretomes predicted in both corn pathogens *S. reilianum* and *U. maydis* are similar to those reported by Schirawski et al. (2010). The corn pathogen study found that many effector candidate genes from both pathogens were orthologous, consequently the number of SSSPs for them were drastically reduced in our study. When the refined secretome of *F. graminearum* was compared to the data by Brown et al. (2012), their 539 out of 574 proteins were included in our 961 secretome. Although we predicted larger secretome than their refined secretome, this is due to the lowered parameter settings for prediction as our parameters were determined based on the effectors listed in Stergiopoulos and de Wit (2009) for kingdom-wide analysis. We also used a greater number of species to cover more diverse taxa and further divided plant pathogens into four groups to perform comprehensive analyses.

The roles of fungal effector proteins regarding lifestyles are previously discussed by Lowe and Howlett (2012) and Lo Presti et al. (2015). These studies suggested that fungi with same lifestyles have similar secretome proportions. The secreted CAZymes also showed similar pattern that biotrophs encode fewer CAZymes than hemibiotrophs and necrotrophs (Zhao et al., 2014; Lo Presti et al., 2015). Although our overall conclusion on refined secretome and CAZymes may be similar with the previous studies, the numbers of SSSPs show lifestyle adaptation, different from secretomes and CAZymes analyses. In addition, secretomes contain not only CAZymes for cell wall degradation and utilization of its components as nutrients but also proteases, lipases, and oxidoreductases for breakdown of other macromolecules, and self-protection and/or pathogenesis. For example, AVR-pita of *M. oryzae* is a zinc metalloprotease that acts as an avirulence factor in its host rice (Zhang and Xu, 2014). The lipase effector FGL1 in *F. graminearum* suppresses callose formation in wheat and is required for host infection (Blümke et al., 2014). Oxidoreductases have been investigated in phytopathogenic fungi for their roles in scavenging plant reactive oxygen species and pathogenicity (Chi et al., 2009). Since their importance in pathogenesis has been established or suggested, we examined the proportions of these enzymes relative to the whole proteome in light of the lifestyle and taxonomic position of individual species. Proteases, lipases, and oxidoreductases seem to be more abundant in plant pathogens, especially in hemibiotrophs and necrotrophs, than the species with different lifestyles. Since the majority of known fungal effector proteins do not possess enzymatic activity, we excluded the above enzyme sets prior to mining SSPs, which include short proteins (≤300 aa) with a signal peptide, but no transmembrane domain or GPI-anchor. Species-specific presence was also analyzed to classify SSPs into SSSPs, which likely act as host-specific effectors. Overall, we found that the size of refined secretomes and SSSPs varies widely between species, but some patterns associated with lifestyles.

In general, phytopathogenic fungi tend to have larger secretomes than non-pathogens. Although, Lowe and Howlett (2012) suggested that animal pathogens generally have a lower proportion of secretome, the sizes of secretomes and proteins of other functions in certain animal pathogens, such as nematophagus and entomopathogenic fungi, were similar to some necrotrophic plant pathogens. This is not too surprising considering that nematophagus and entomopathogenic fungi secrete diverse proteins to facilitate infection and consumption of hosts (Andersson et al., 2013; Staats et al., 2014). The host-specific animal pathogens that co-evolved with hosts for longer period of time may have large secretome, yet smaller than those of phytopathogens. Although symbionts intimately interact with plant hosts, their secretomes are smaller than those of pathogens, a pattern that was found in previous studies on symbionts such as *L. bicolor* (Martin et al., 2008) and *Tuber melanosporum* (Martin et al., 2010). This is due to the reduced number of CAZymes compared to necrotrophs and hemibiotrophs. Biotrophs encode smaller sets of CAZymes, but larger secretomes than non-pathogens mainly due to their abundant SSSPs. This reflects the lifestyle of biotrophs which causes minimal damages to the hosts to maintain a long-term feeding relationship. Not surprisingly, reduced numbers of SSSPs are observed among crop-infecting necrotrophs. However, wood-decaying necrotrophs conspicuously possess a similar level of SSSPs to hemibiotrophs. Their roles of SSSPs in wood-decay remain unclear. Within saprotrophs, fruit-body forming fungi and fungi displaying antifungal activities encode greater numbers of SSSPs than yeasts and extremophiles. However, the number of SSSPs has no correlation with host range within necrotrophs, types of rot for wood-decaying necrotrophs, or types of association for symbionts. As illustrated by *Neurospora crassa*, a saprotrophic fungus that has been reported to be associated with pine trees in harsh conditions and even pathogenic (Kuo et al., 2014), fungal lifestyles may not be a fixed attribute, but is changeable depending on environmental conditions.

Another concern is that the number of small proteins could be affected by the minimum protein size used to annotate each genome. However, we strictly used the annotated data associated with published genomes, thus we can assume that the numbers of annotated proteins were comparable to each other. In addition, the majority of genome data were also from JGI and Broad Institute, which followed the conventional JGI annotation process and Broad Gene Finding Methods, respectively. If the minimum cutoff length for gene prediction was stated, only short peptides without EST support were eliminated. Since, the majority of fungal genome studies did not show the cutoff size for protein-coding gene prediction, we analyzed and compared the length distribution of the annotated proteomes for validation (the last column of Supplementary Table S1). In result, no parameters were possibly used for 50 species and additional 72 species with the cutoff of 30 aa. The rest 14 species contained proteins with length at least 50 aa. The number of short proteins in the first category was extremely high for a few species such as *C. fulvum* and *P. confluens*. However, only the proteins secreted with canonical pathways were considered in this analysis, which means the minimum size of protein is bound to the length of signal peptide that is 15 to 40 aa (Choo et al., 2005). Although many short proteins were annotated using no or very short cutoff, they were eliminated if the signal peptide was absent. Overall, we believe that the numbers of proteins are generally comparable like in other secretome studies.

Many genes coding candidate effectors in plant pathogens and virulence-associated genes of animal pathogens have been reported to reside in gene-sparse, AT-rich, and telomere-proximal genomic regions (Schmidt and Panstruga, 2011). However, genomic distribution patterns of SSSP-coding genes did not display similar trends, with the exception of those in the wood-decaying necrotrophs. Most SSSP-coding genes in these fungi were found in genomic regions with low gene numbers. A previous study reported that 23% of the proteins encoded by these wood-decaying fungal species are unique (Riley et al., 2014), indicating that the SSSP-coding genes recently arose from non-coding sequences as suggested by Carvunis Model (Carvunis et al., 2012). However, there are still many SSSP-coding genes located in AT-rich regions. Therefore, the genomic context could be considered for prioritizing validation of effector functions. Although effector proteins are often thought to be species-specific due to co-evolution with the host, there are cases of conserved effectors within related species. For example, the *C. fulvum* effector Ecp2 (Stergiopoulos et al., 2012) and the cerato-platanin proteins (Chen et al., 2013) are conserved only within Ascomycota and Basidiomycota. Moreover, the clustering analysis of CSSPs showed that none of them seem to be conserved across the fungal kingdom, indicating that they are typically limited to specific genera. However, some CSSPs of *B. graminis*, e.g., BEC1040, BEC1019, and BEC1005, were found in fungi having different lifestyles other than biotroph. These observations were further supported by the facts that these proteins are involved in fungal development, and resemble metalloprotease and glucanase, respectively (Pliego et al., 2013). Taken together, these suggest that the numbers of CSSPs may not be correlated with fungal lifestyles.

In conclusion, different secretome components reflect lifestyle-associated genomic adaptations in fungi. Results from this comparative study provide new insights into the genetic basis and molecular evolution of fungal lifestyles and also establish a solid foundation for future discovery and functional validation of effectors.

## Author contributions

KK and YL designed this project. KK, JJ, HS and GC performed computational analyses. JC and KC provided the secretome and the genome data for analyses. KK, JJ, SK, and YL wrote the manuscript. SK and YL supervised the research. All authors read and approved the manuscript.

### Conflict of interest statement

The authors declare that the research was conducted in the absence of any commercial or financial relationships that could be construed as a potential conflict of interest.

## Abbreviations

SSPs: small secreted proteins
SSSPs: species-specific SSPs
CSSPs: conserved SSPs.

## References

[B1] Andersson K. M. Meerupati T. Levander F. Friman E. Ahrén D. Tunlid A. (2013). Proteome of the nematode-trapping cells of the fungus *Monacrosporium haptotylum*. Appl. Environ. Microbiol. 79, 4993–5004. 10.1128/Aem.01390-13 23770896 PMC3754708

[B2] Birch P. R. J. Boevink P. C. Gilroy E. M. Hein I. Pritchard L. Whisson S. C. (2008). Oomycete RXLR effectors: delivery, functional redundancy and durable disease resistance. Curr. Opin. Plant Biol. 11, 373–379. 10.1016/j.pbi.2008.04.005 18511334

[B3] Blümke A. Falter C. Herrfurth C. Sode B. Bode R. Schäfer W.. (2014). Secreted fungal effector lipase releases free fatty acids to inhibit innate immunity-related callose formation during wheat head infection. Plant Physiol. 165, 346–358. 10.1104/pp.114.236737 24686113 PMC4012593

[B4] Brown N. A. Antoniw J. Hammond-Kosack K. E. (2012). The predicted secretome of the plant pathogenic fungus *Fusarium graminearum*: a refined comparative analysis. PLoS ONE 7:e33731. 10.1371/journal.pone.0033731 22493673 PMC3320895

[B5] Cao W. Maruyama J. Kitamoto K. Sumikoshi K. Terada T. Nakamura S.. (2009). Using a new GPI-anchored-protein identification system to mine the protein databases of *Aspergillus fumigatus, Aspergillus nidulans*, and *Aspergillus oryzae*. J. Gen. Appl. Microbiol. 55, 381–393. 10.2323/jgam.55.381 19940384

[B6] Carvunis A. R. Rolland T. Wapinski I. Calderwood M. A. Yildirim M. A. Simonis N.. (2012). Proto-genes and *de novo* gene birth. Nature 487, 370–374. 10.1038/nature11184 22722833 PMC3401362

[B7] Chen H. X. Kovalchuk A. Keriö S. Asiegbu F. O. (2013). Distribution and bioinformatic analysis of the cerato-platanin protein family in Dikarya. Mycologia 105, 1479–1488. 10.3852/13-115 23928425

[B8] Chen K. Durand D. Farach-Colton M. (2000). NOTUNG: a program for dating gene duplications and optimizing gene family trees. J. Comput. Biol. 7, 429–447. 10.1089/106652700750050871 11108472

[B9] Cheng Q. Wang H. Xu B. Zhu S. Hu L. Huang M. (2014). Discovery of a novel small secreted protein family with conserved N-terminal IGY motif in Dikarya fungi. BMC Genomics 15:1151. 10.1186/1471-2164-15-1151 25526808 PMC4367982

[B10] Chi M. H. Park S. Y. Kim S. Lee Y. H. (2009). A novel pathogenicity gene is required in the rice blast fungus to suppress the basal defenses of the host. PLoS Pathog. 5:e1000401. 10.1371/journal.ppat.1000401 19390617 PMC2668191

[B11] Choi J. Cheong K. Jung K. Jeon J. Lee G. W. Kang S.. (2013). CFGP 2.0: a versatile web-based platform for supporting comparative and evolutionary genomics of fungi and oomycetes. Nucleic Acids Res. 41, D714–D719. 10.1093/nar/gks1163 23193288 PMC3531191

[B12] Choi J. Détry N. Kim K. T. Asiegbu F. O. Valkonen J. P. T. Lee Y. H. (2014). fPoxDB: fungal peroxidase database for comparative genomics. BMC Microbiol. 14:117. 10.1186/1471-2180-14-117 24885079 PMC4029949

[B13] Choi J. Park J. Kim D. Jung K. Kang S. Lee Y. H. (2010). Fungal secretome database: integrated platform for annotation of fungal secretomes. BMC Genomics 11:105. 10.1186/1471-2164-11-105 20146824 PMC2836287

[B14] Choo K. H. Tan T. W. Ranganathan S. (2005). SPdb – a signal peptide database. BMC Bioinformatics 6:249. 10.1186/1471-2105-6-249 16221310 PMC1276010

[B15] Cornelis G. R. Van Gijsegem F. (2000). Assembly and function of type III secretory systems. Annu. Rev. Microbiol. 54, 735–774. 10.1146/annurev.micro.54.1.735 11018143

[B16] Deller S. Hammond-Kosack K. E. Rudd J. J. (2011). The complex interactions between host immunity and non-biotrophic fungal pathogens of wheat leaves. J. Plant Physiol. 168, 63–71. 10.1016/j.jplph.2010.05.024 20688416

[B17] Dodds P. N. Rathjen J. P. (2010). Plant immunity: towards an integrated view of plant-pathogen interactions. Nat. Rev. Genet. 11, 539–548. 10.1038/nrg2812 20585331

[B18] Duplessis S. Cuomo C. A. Lin Y. C. Aerts A. Tisserant E. Veneault-Fourrey C.. (2011). Obligate biotrophy features unraveled by the genomic analysis of rust fungi. Proc. Natl. Acad. Sci. U.S.A. 108, 9166–9171. 10.1073/pnas.1019315108 21536894 PMC3107277

[B19] Enright A. J. Van Dongen S. Ouzounis C. A. (2002). An efficient algorithm for large-scale detection of protein families. Nucleic Acids Res. 30, 1575–1584. 10.1093/nar/30.7.1575 11917018 PMC101833

[B20] Fischer M. Pleiss J. (2003). The lipase engineering database: a navigation and analysis tool for protein families. Nucleic Acids Res. 31, 319–321. 10.1093/nar/gkg015 12520012 PMC165462

[B21] Giraldo M. C. Valent B. (2013). Filamentous plant pathogen effectors in action. Nat. Rev. Microbiol. 11, 800–814. 10.1038/nrmicro3119 24129511

[B22] Girard V. Dieryckx C. Job C. Job D. (2013). Secretomes: the fungal strike force. Proteomics 13, 597–608. 10.1002/pmic.201200282 23349114

[B23] Godfrey D. Böhlenius H. Pedersen C. Zhang Z. Emmersen J. Thordal-Christensen H. (2010). Powdery mildew fungal effector candidates share N-terminal Y/F/WxC-motif. BMC Genomics 11:317. 10.1186/1471-2164-11-317 20487537 PMC2886064

[B24] Gourion B. Berrabah F. Ratet P. Stacey G. (2015). Rhizobium-legume symbioses: the crucial role of plant immunity. Trends Plant Sci. 20, 186–194. 10.1016/j.tplants.2014.11.008 25543258

[B25] Guyon K. Balagué C. Roby D. Raffaele S. (2014). Secretome analysis reveals effector candidates associated with broad host range necrotrophy in the fungal plant pathogen *Sclerotinia sclerotiorum*. BMC Genomics 15:336. 10.1186/1471-2164-15-336 24886033 PMC4039746

[B26] Hacquard S. Joly D. L. Lin Y. C. Tisserant E. Feau N. Delaruelle C.. (2012). A comprehensive analysis of genes encoding small secreted proteins identifies candidate effectors in *Melampsora larici-populina* (poplar leaf rust). Mol. Plant Microbe Interact. 25, 279–293. 10.1094/MPMI-09-11-0238 22046958

[B27] Hall B. G. (2013). Building phylogenetic trees from molecular data with MEGA. Mol. Biol. Evol. 30, 1229–1235. 10.1093/molbev/mst012 23486614

[B28] Horton P. Park K. J. Obayashi T. Fujita N. Harada H. Adams-Collier C. J.. (2007). WoLF PSORT: protein localization predictor. Nucleic Acids Res. 35, W585–W587. 10.1093/nar/gkm259 17517783 PMC1933216

[B29] Jones J. D. G. Dangl J. L. (2006). The plant immune system. Nature 444, 323–329. 10.1038/nature05286 17108957

[B30] Käll L. Krogh A. Sonnhammer E. L. L. (2004). A combined transmembrane topology and signal peptide prediction method. J. Mol. Biol. 338, 1027–1036. 10.1016/j.jmb.2004.03.016 15111065

[B31] Kim S. Park J. Park S. Y. Mitchell T. K. Lee Y. H. (2010). Identification and analysis of *in planta* expressed genes of *Magnaporthe oryzae*. BMC Genomics 11:104. 10.1186/1471-2164-11-104 20146797 PMC2832786

[B32] Kloppholz S. Kuhn H. Requena N. (2011). A secreted fungal effector of *Glomus intraradices* promotes symbiotic biotrophy. Curr. Biol. 21, 1204–1209. 10.1016/j.cub.2011.06.044 21757354

[B33] Krijger J. J. Thon M. R. Deising H. B. Wirsel S. G. R. (2014). Compositions of fungal secretomes indicate a greater impact of phylogenetic history than lifestyle adaptation. BMC Genomics 15:722. 10.1186/1471-2164-15-722 25159997 PMC4161775

[B34] Kuo H. C. Hui S. Choi J. Asiegbu F. O. Valkonen J. P. T. Lee Y. H. (2014). Secret lifestyles of *Neurospora crassa*. Sci. Rep. 4:5135. 10.1038/srep05135 24875794 PMC4038807

[B35] Lo Presti L. Lanver D. Schweizer G. Tanaka S. Liang L. Tollot M.. (2015). Fungal effectors and plant susceptibility. Annu. Rev. Plant Biol. 66, 513–545. 10.1146/annurev-arplant-043014-114623 25923844

[B36] Lowe R. G. T. Howlett B. J. (2012). Indifferent, affectionate, or deceitful: lifestyles and secretomes of fungi. PLoS Pathog. 8:e1002515. 10.1371/journal.ppat.1002515 22396640 PMC3291654

[B37] Martin F. Aerts A. Ahrén D. Brun A. Danchin E. G. J. Duchaussoy F.. (2008). The genome of *Laccaria bicolor* provides insights into mycorrhizal symbiosis. Nature 452, 88–92. 10.1038/nature06556 18322534

[B38] Martin F. Kohler A. Murat C. Balestrini R. Coutinho P. M. Jaillon O.. (2010). Perigord black truffle genome uncovers evolutionary origins and mechanisms of symbiosis. Nature 464, 1033–1038. 10.1038/nature08867 20348908

[B39] Meinken J. Asch D. K. Neizer-Ashun K. A. Chang G. H. Cooper C. R. Jr. Min X. J. (2014). FunSecKB2: a fungal protein subcellular location knowledgebase. Comput. Mol. Biol. 4, 1–17. 10.5376/cmb.2014.04.0007

[B40] Plett J. M. Kemppainen M. Kale S. D. Kohler A. Legué V. Brun A.. (2011). A secreted effector protein of *Laccaria bicolor* is required for symbiosis development. Curr. Biol. 21, 1197–1203. 10.1016/j.cub.2011.05.033 21757352

[B41] Pliego C. Nowara D. Bonciani G. Gheorghe D. M. Xu R. Surana P.. (2013). Host-induced gene silencing in barley powdery mildew reveals a class of ribonuclease-like effectors. Mol. Plant Microbe Interact. 26, 633–642. 10.1094/MPMI-01-13-0005-R 23441578

[B42] Rafiqi M. Ellis J. G. Ludowici V. A. Hardham A. R. Dodds P. N. (2012). Challenges and progress towards understanding the role of effectors in plant-fungal interactions. Curr. Opin. Plant Biol. 15, 477–482. 10.1016/j.pbi.2012.05.003 22658704

[B43] Rawlings N. D. Waller M. Barrett A. J. Bateman A. (2014). MEROPS: the database of proteolytic enzymes, their substrates and inhibitors. Nucleic Acids Res. 42, D503–D509. 10.1093/nar/gkt953 24157837 PMC3964991

[B44] Rep M. (2005). Small proteins of plant-pathogenic fungi secreted during host colonization. FEMS Microbiol. Lett. 253, 19–27. 10.1016/j.femsle.2005.09.014 16216445

[B45] Riley R. Salamov A. A. Brown D. W. Nagy L. G. Floudas D. Held B. W.. (2014). Extensive sampling of basidiomycete genomes demonstrates inadequacy of the white-rot/brown-rot paradigm for wood decay fungi. Proc. Natl. Acad. Sci. U.S.A. 111, 14959–14959. 10.1073/pnas.1418116111 24958869 PMC4103376

[B46] Rogers L. M. Flaishman M. A. Kolattukudy P. E. (1994). Cutinase gene disruption in *Fusarium solani* f sp pisi decreases its virulence on pea. Plant Cell 6, 935–945. 10.1105/tpc.6.7.935 8069105 PMC160490

[B47] Rovenich H. Boshoven J. C. Thomma B. P. (2014). Filamentous pathogen effector functions: of pathogens, hosts and microbiomes. Curr. Opin. Plant Biol. 20, 96–103. 10.1016/j.pbi.2014.05.001 24879450

[B48] Santhanam P. Thomma B. P. (2013). *Verticillium dahliae* Sge1 differentially regulates expression of candidate effector genes. Mol. Plant Microbe Interact. 26, 249–256. 10.1094/Mpmi-08-12-0198-R 22970788

[B49] Saunders D. G. O. Win J. Cano L. M. Szabo L. J. Kamoun S. Raffaele S. (2012). Using hierarchical clustering of secreted protein families to classify and rank candidate effectors of rust fungi. PLoS ONE 7:e29847. 10.1371/journal.pone.0029847 22238666 PMC3253089

[B50] Schirawski J. Mannhaupt G. Münch K. Brefort T. Schipper K. Doehlemann G.. (2010). Pathogenicity determinants in smut fungi revealed by genome comparison. Science 330, 1546–1548. 10.1126/science.1195330 21148393

[B51] Schmidt S. M. Panstruga R. (2011). Pathogenomics of fungal plant parasites: what have we learnt about pathogenesis? Curr. Opin. Plant Biol. 14, 392–399. 10.1016/j.pbi.2011.03.006 21458359

[B52] Seidl M. F. Faino L. Shi-Kunne X. van den Berg G. C. M. Bolton M. D. Thomma B. P. (2015). The genome of the saprophytic fungus *Verticillium tricorpus* reveals a complex effector repertoire resembling that of its pathogenic relatives. Mol. Plant Microbe Interact. 28, 362–373. 10.1094/Mpmi-06-14-0173-R 25208342

[B53] Soto M. J. Sanjuán J. Olivares J. (2006). Rhizobia and plant-pathogenic bacteria: common infection weapons. Microbiology 152, 3167–3174. 10.1099/mic.0.29112-0 17074888

[B54] Spanu P. D. Abbott J. C. Amselem J. Burgis T. A. Soanes D. M. Stüber K.. (2010). Genome expansion and gene loss in powdery mildew fungi reveal tradeoffs in extreme parasitism. Science 330, 1543–1546. 10.1126/science.1194573 21148392

[B55] Sreedhar L. Kobayashi D. Y. Bunting T. E. Hillman B. I. Belanger F. C. (1999). Fungal proteinase expression in the interaction of the plant pathogen *Magnaporthe poae* with its host. Gene 235, 121–129. 10.1016/S0378-1119(99)00201-2 10415340

[B56] Staats C. C. Junges A. Guedes R. L. M. Thompson C. E. de Morais G. L. Boldo J. T.. (2014). Comparative genome analysis of entomopathogenic fungi reveals a complex set of secreted proteins. BMC Genomics 15:822. 10.1186/1471-2164-15-822 25263348 PMC4246632

[B57] Stergiopoulos I. de Wit P. J. (2009). Fungal effector proteins. Annu. Rev. Phytopathol. 47, 233–263. 10.1146/annurev.phyto.112408.132637 19400631

[B58] Stergiopoulos I. Kourmpetis Y. A. I. Slot J. C. Bakker F. T. De Wit P. J. Rokas A. (2012). *In silico* characterization and molecular evolutionary analysis of a novel superfamily of fungal effector proteins. Mol. Biol. Evol. 29, 3371–3384. 10.1093/molbev/mss143 22628532

[B59] Tisserant E. Kohler A. Dozolme-Seddas P. Balestrini R. Benabdellah K. Colard A.. (2012). The transcriptome of the arbuscular mycorrhizal fungus *Glomus intraradices* (DAOM 197198) reveals functional tradeoffs in an obligate symbiont. New Phytol. 193, 755–769. 10.1111/j.1469-8137.2011.03948.x 22092242

[B60] Tisserant E. Malbreil M. Kuo A. Kohler A. Symeonidi A. Balestrini R.. (2013). Genome of an arbuscular mycorrhizal fungus provides insight into the oldest plant symbiosis. Proc. Natl. Acad. Sci. U.S.A. 110, 20117–20122. 10.1073/pnas.1313452110 24277808 PMC3864322

[B61] Urban M. Pant R. Raghunath A. Irvine A. G. Pedro H. Hammond-Kosack K. E. (2015). The Pathogen-Host Interactions database (PHI-base): additions and future developments. Nucleic Acids Res. 43, D645–D655. 10.1093/nar/gku1165 25414340 PMC4383963

[B62] van Esse H. P. Bolton M. D. Stergiopoulos I. de Wit P. J. Thomma B. P. (2007). The chitin-binding *Cladosporium fulvum* effector protein Avr4 is a virulence factor. Mol. Plant Microbe Interact. 20, 1092–1101. 10.1094/MPMI-20-9-1092 17849712

[B63] Vargas W. A. Mandawe J. C. Kenerley C. M. (2009). Plant-derived sucrose is a key element in the symbiotic association between *Trichoderma virens* and maize plants. Plant Physiol. 151, 792–808. 10.1104/pp.109.141291 19675155 PMC2754623

[B64] Whisson S. C. Boevink P. C. Moleleki L. Avrova A. O. Morales J. G. Gilroy E. M.. (2007). A translocation signal for delivery of oomycete effector proteins into host plant cells. Nature 450, 115–118. 10.1038/nature06203 17914356

[B65] Xu Z. Hao B. (2009). CVTree update: a newly designed phylogenetic study platform using composition vectors and whole genomes. Nucleic Acids Res. 37, W174–W178. 10.1093/nar/gkp278 19398429 PMC2703908

[B66] Yin Y. Mao X. Yang J. Chen X. Mao F. Xu Y. (2012). dbCAN: a web resource for automated carbohydrate-active enzyme annotation. Nucleic Acids Res. 40, W445–W451. 10.1093/nar/gks479 22645317 PMC3394287

[B67] Zamioudis C. Pieterse C. M. J. (2012). Modulation of host immunity by beneficial microbes. Mol. Plant Microbe Interact. 25, 139–150. 10.1094/Mpmi-06-11-0179 21995763

[B68] Zhang S. Xu J. R. (2014). Effectors and effector delivery in *Magnaporthe oryzae*. PLoS Pathog. 10:e1003826. 10.1371/journal.ppat.1003826 24391496 PMC3879361

[B69] Zhao Z. Liu H. Wang C. Xu J. R. (2014). Comparative analysis of fungal genomes reveals different plant cell wall degrading capacity in fungi. BMC Genomics 15:6. 10.1186/1471-2164-15-6 24422981 PMC3893384

[B70] Zuccaro A. Lahrmann U. Güldener U. Langen G. Pfiffi S. Biedenkopf D.. (2011). Endophytic life strategies decoded by genome and transcriptome analyses of the mutualistic root symbiont *Piriformospora indica*. PLoS Pathog. 7:e1002290. 10.1371/journal.ppat.1002290 22022265 PMC3192844

