# Supplementary figures and images for: Kingdom-Wide Analysis of Fungal Small Secreted Proteins (SSPs) Reveals their Potential Role in Host Association
Source: Front Plant Sci. 2016 Feb 19;7:186. doi: 10.3389/fpls.2016.00186 (PMC4759460; doi:10.3389/fpls.2016.00186)

Figure S2 | Phylogenetic tree for 136 species and their refined secretome.

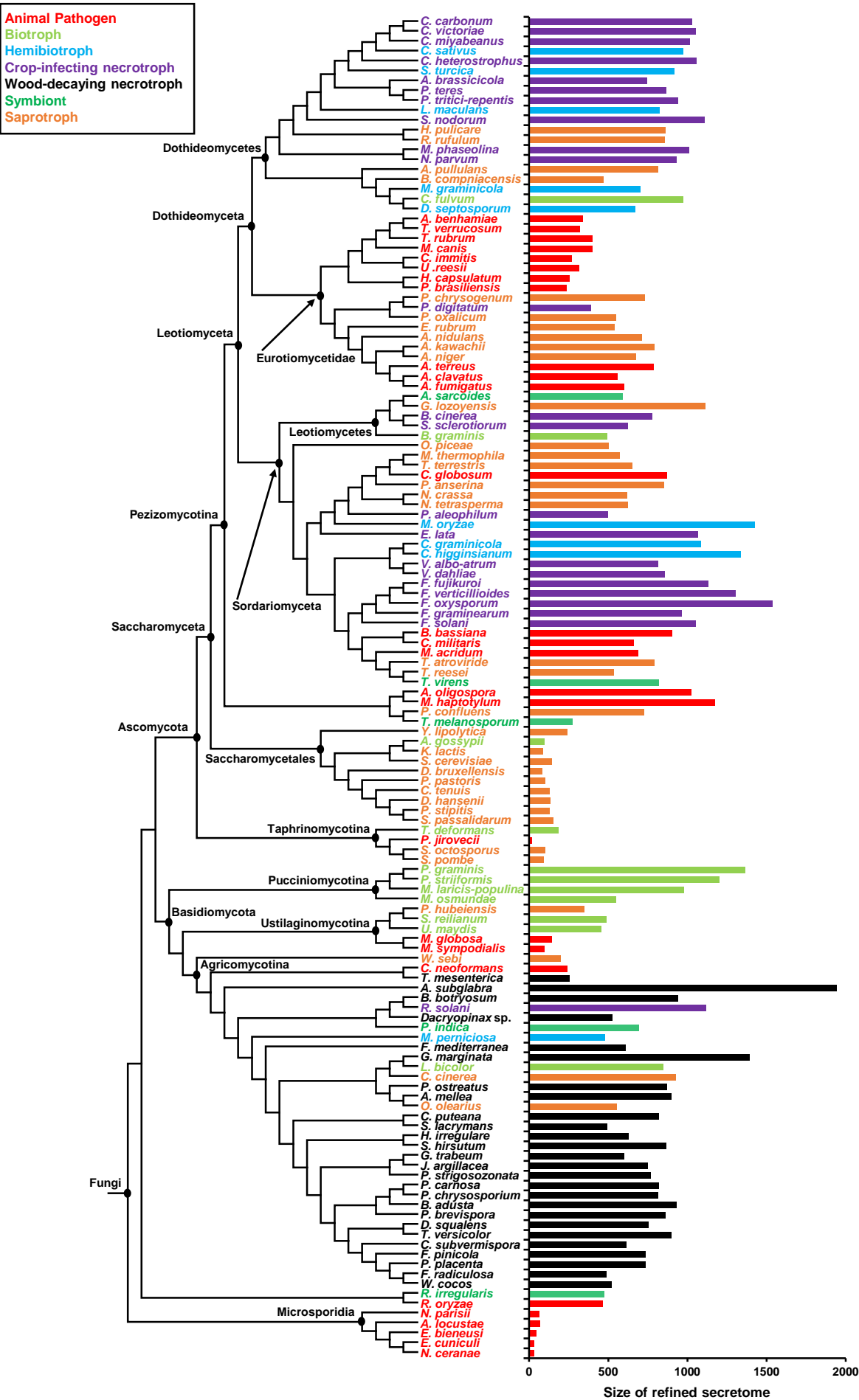

Supplement: Supplementary file 6 [file Presentation2.PDF]
