# Supplementary material for: Kingdom-Wide Analysis of Fungal Small Secreted Proteins (SSPs) Reveals their Potential Role in Host Association
Source: Front Plant Sci. 2016 Feb 19;7:186. doi: 10.3389/fpls.2016.00186 (PMC4759460; doi:10.3389/fpls.2016.00186)
Supplement: Supplementary file 7 [file Presentation3.PDF]

Figure S3 | Protein length distributions within the proteins of other functions.

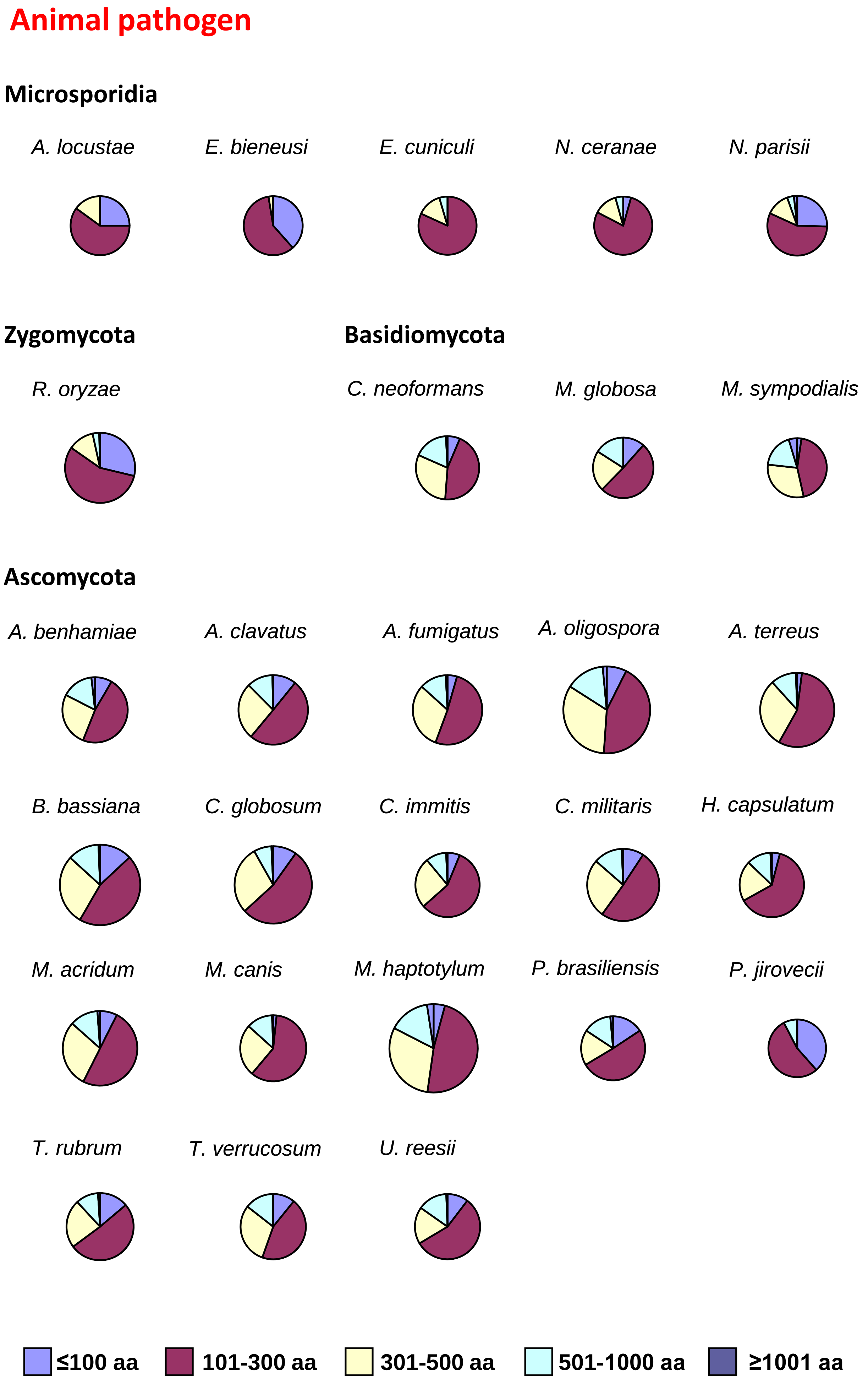

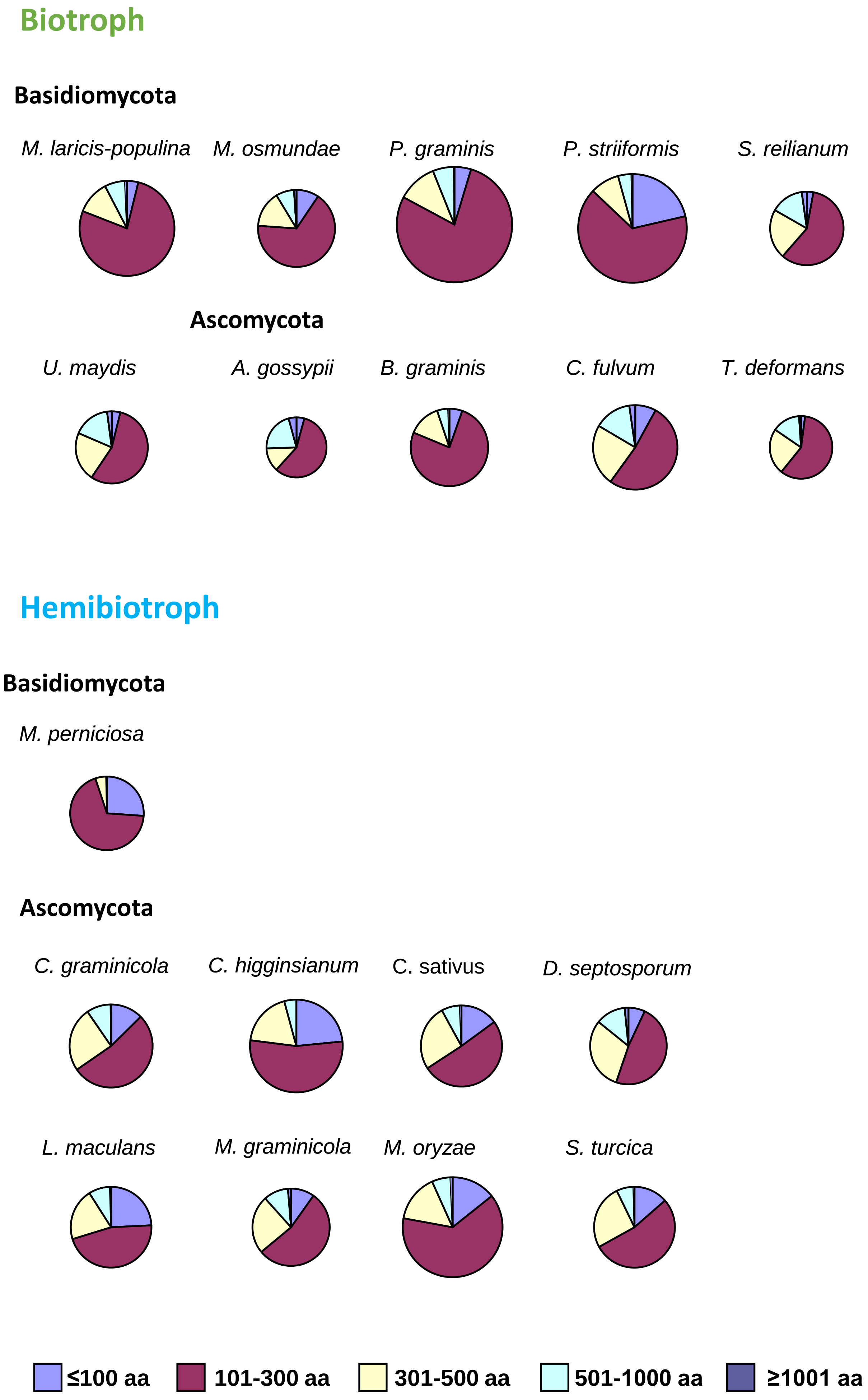

Biotroph
Basidiomycota
M. laricis-populina
M. osmundae
P. graminis
P. striiformis
S. reilianum
Ascomycota
U. maydis
A. gossypii
B. graminis
C. fulvum
T. deformans
Hemibiotroph
Basidiomycota
M. perniciosa
Ascomycota
C. graminicola
C. higginsianum
C. sativus
D. septosporum
L. maculans
M. graminicola
M. oryzae
S. turcica
≤100 aa
101-300 aa
301-500 aa
501-1000 aa
≥1001 aa

Crop-infecting necrotroph

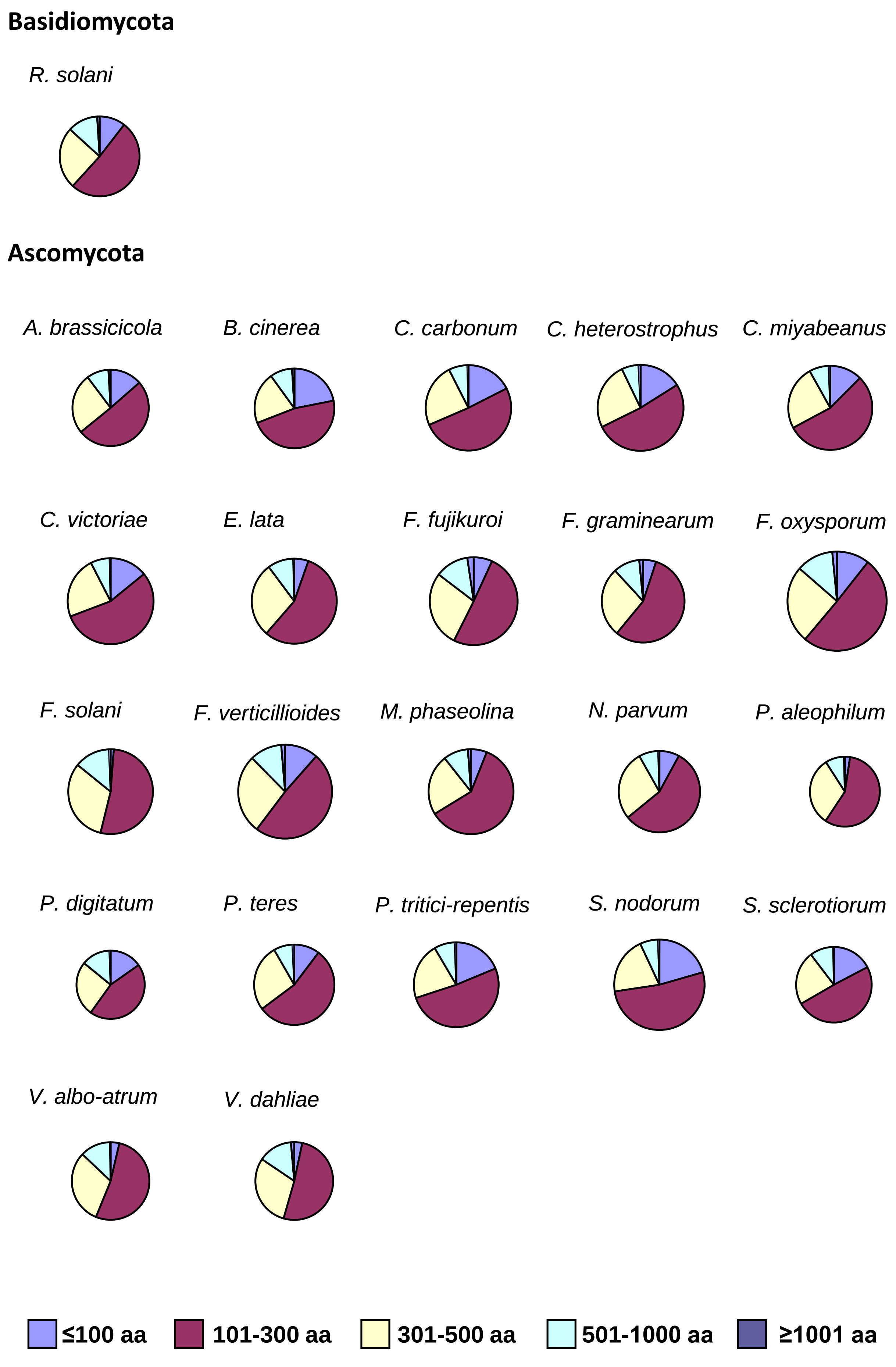

Wood-decaying necrotroph

Basidiomycota

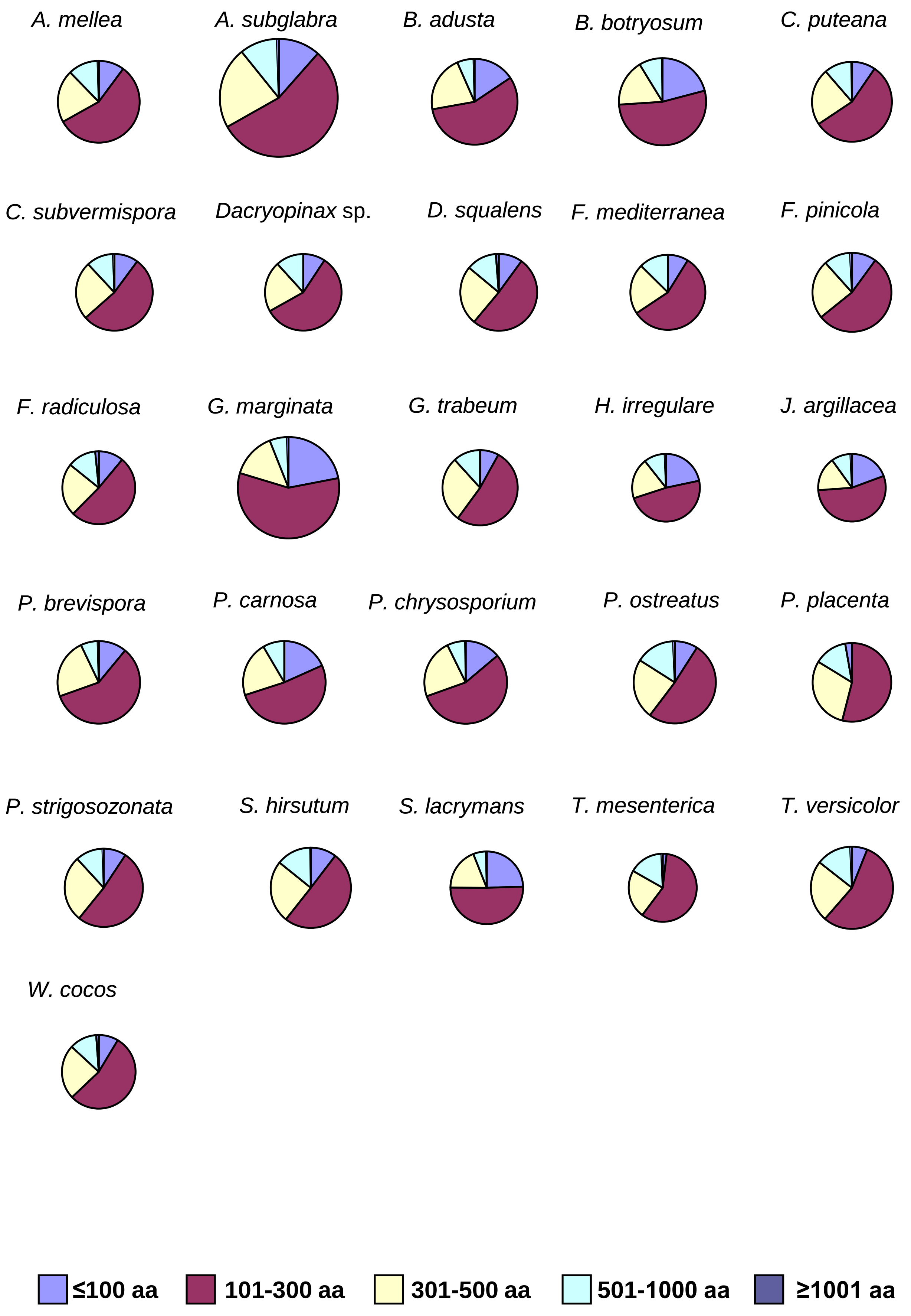

Saprotroph

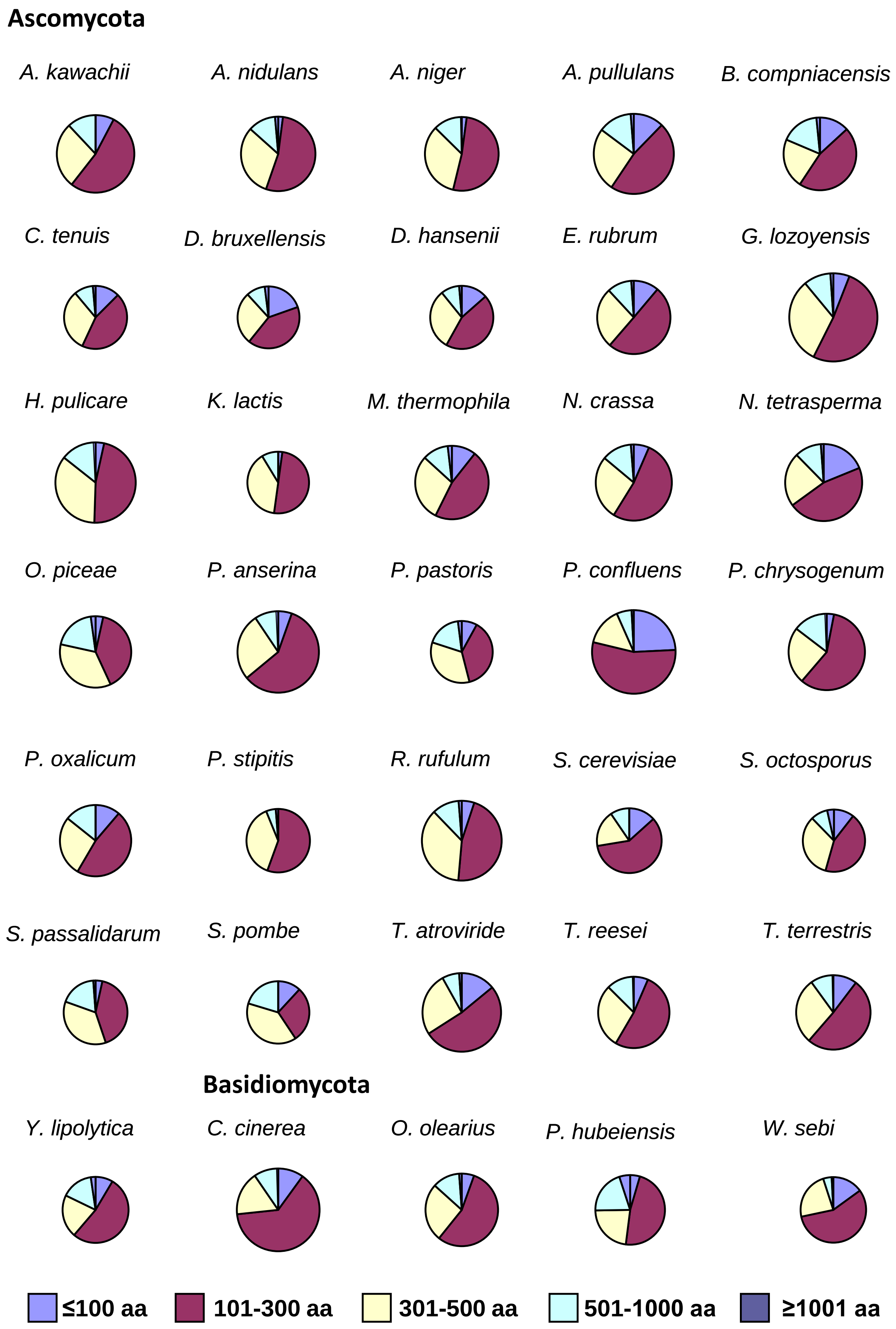
Ascomycota
A. kawachii
A. nidulans
A. niger
A. pullulans
B. compniacensis
C. tenuis
D. bruxellensis
D. hansenii
E. rubrum
G. lozoyensis
H. pulicare
K. lactis
M. thermophila
N. crassa
N. tetrasperma
O. piceae
P. anserina
P. pastoris
P. confluens
P. chrysogenum
P. oxalicum
P. stipitis
R. rufulum
S. cerevisiae
S. octosporus
S. passalidarum
S. pombe
T. atroviride
T. reesei
T. terrestris
Basidiomycota
Y. lipolytica
C. cinerea
O. olearius
P. hubeiensis
W. sebi
≤100 aa
101-300 aa
301-500 aa
501-1000 aa
≥1001 aa

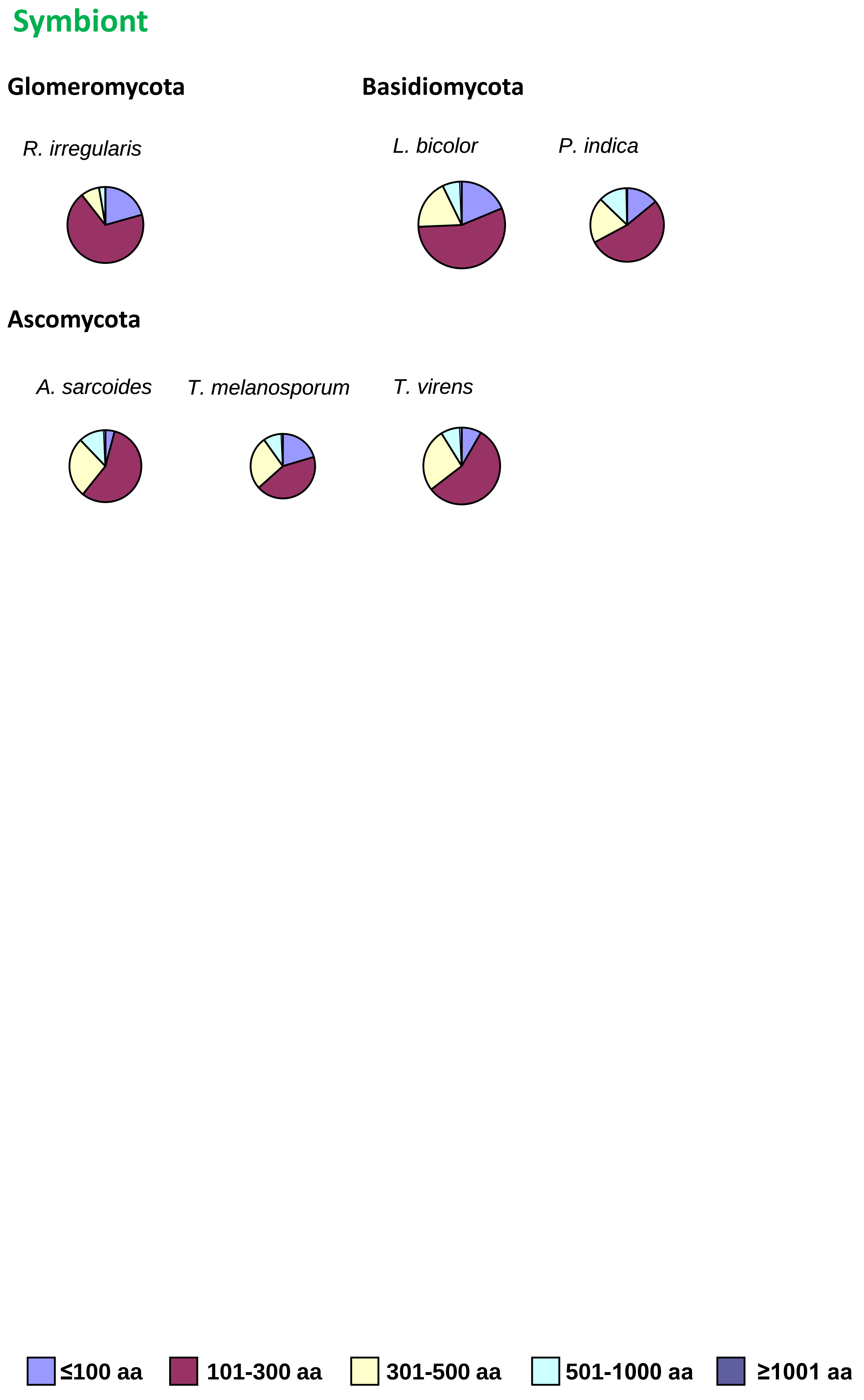
Symbiont
Glomeromycota
R. irregularis
Basidiomycota
L. bicolor
P. indica
Ascomycota
A. sarcoides
T. melanosporum
T. virens
≤100 aa
101-300 aa
301-500 aa
501-1000 aa
≥1001 aa
