# Supplementary material for: Kingdom-Wide Analysis of Fungal Small Secreted Proteins (SSPs) Reveals their Potential Role in Host Association
Source: Front Plant Sci. 2016 Feb 19;7:186. doi: 10.3389/fpls.2016.00186 (PMC4759460; doi:10.3389/fpls.2016.00186)
Supplement: Supplementary file 8 [file Presentation4.PDF]

Figure S4 | Species-specific proteins in the proteins of other functions.

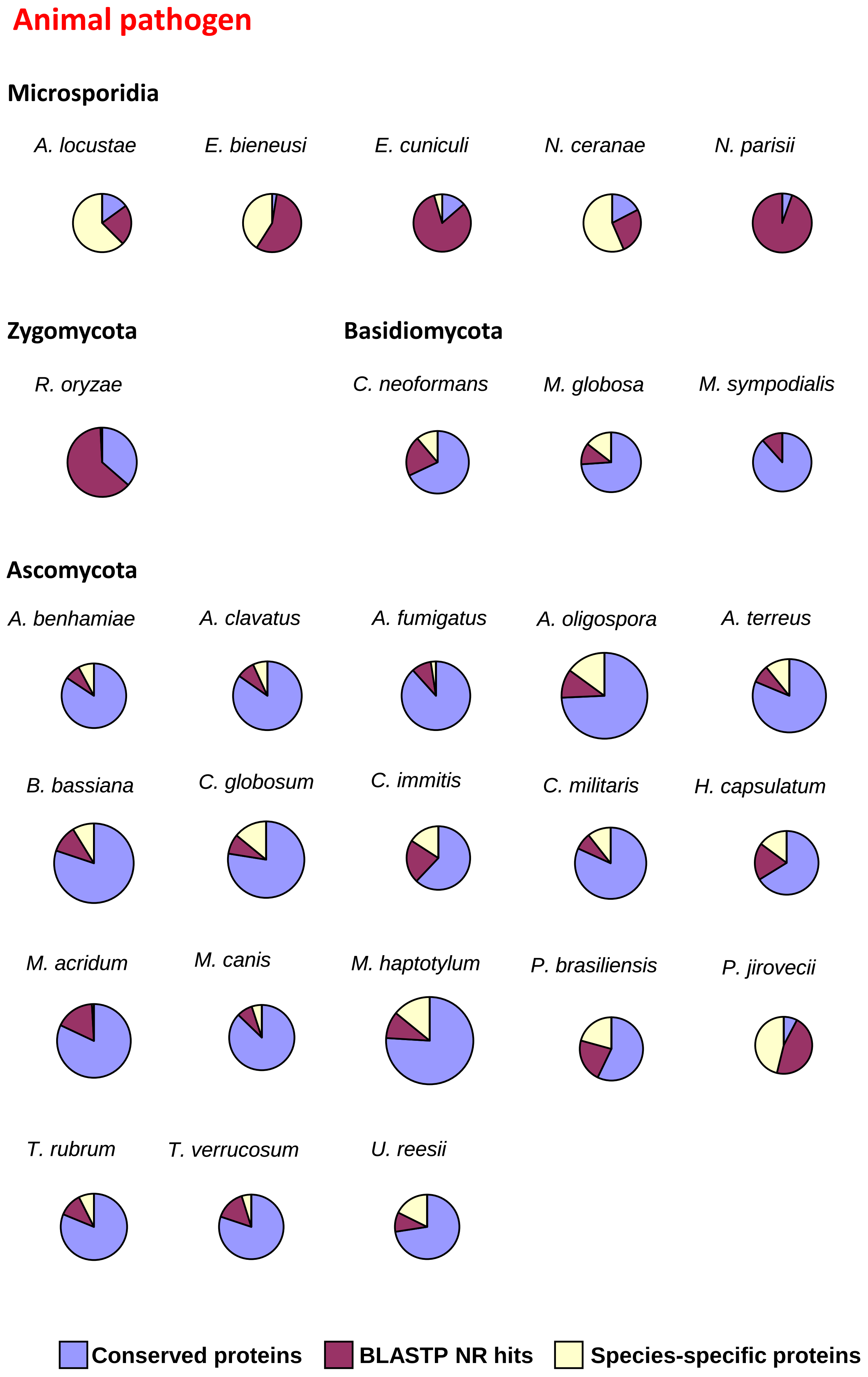

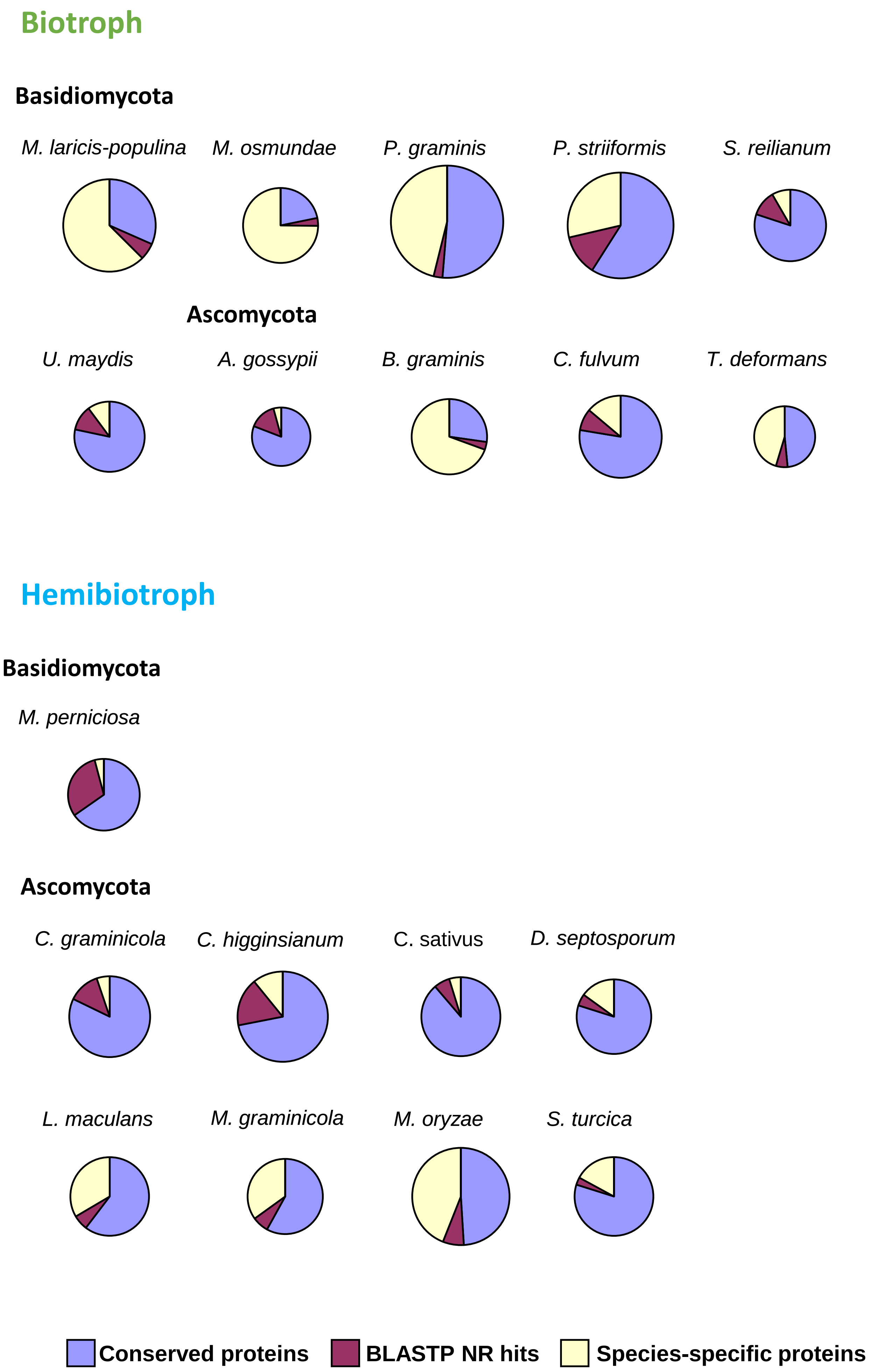
Biotroph
Basidiomycota
M. laricis-populina
M. osmundae
P. graminis
P. striiformis
S. reilianum
Ascomycota
U. maydis
A. gossypii
B. graminis
C. fulvum
T. deformans
Hemibiotroph
Basidiomycota
M. perniciosa
Ascomycota
C. graminicola
C. higginsianum
C. sativus
D. septosporum
L. maculans
M. graminicola
M. oryzae
S. turcica
Conserved proteins
BLASTP NR hits
Species-specific proteins

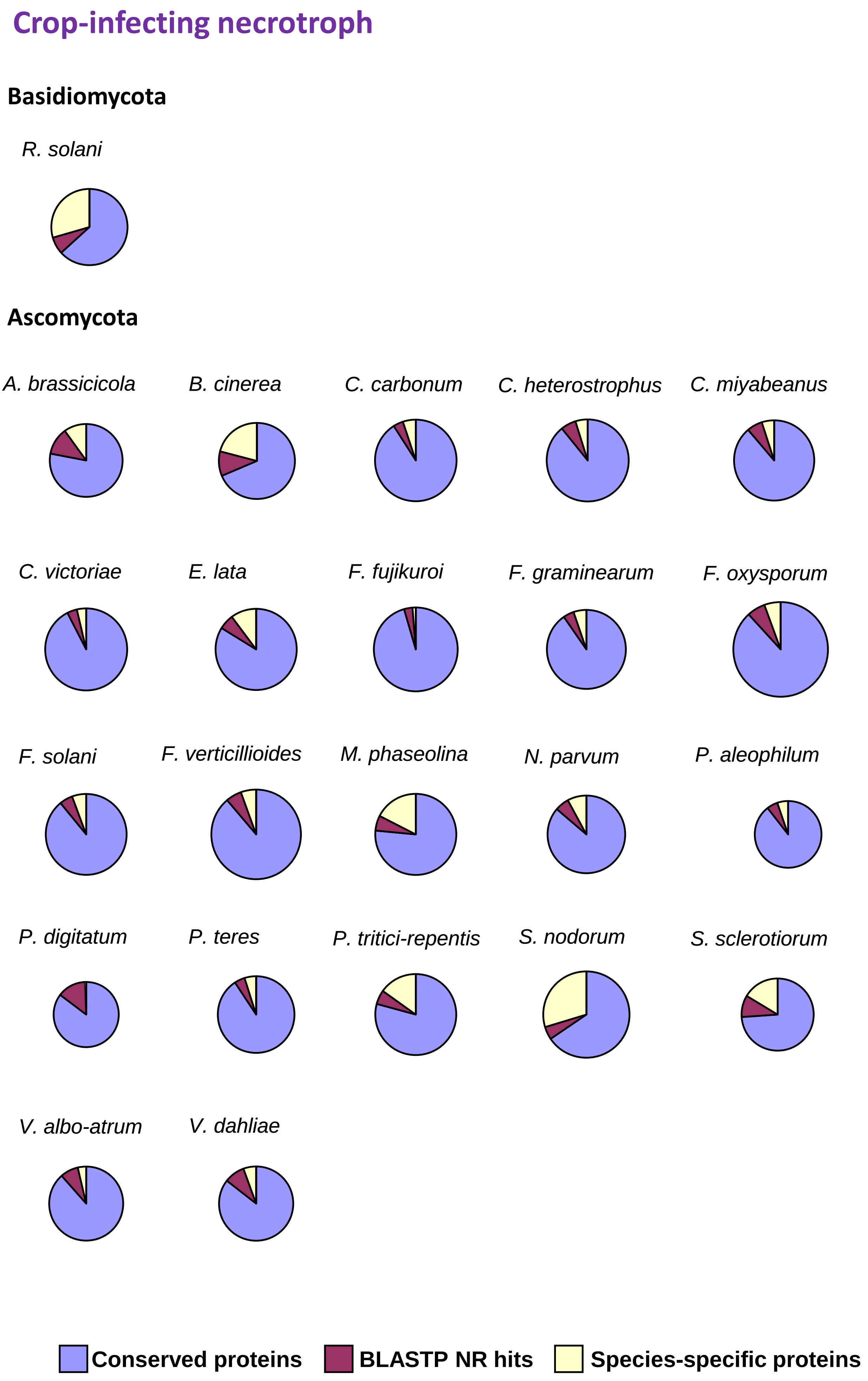
Crop-infecting necrotroph
Basidiomycota
R. solani
Ascomycota
A. brassicicola
B. cinerea
C. carbonum
C. heterostrophus
C. miyabeanus
C. victoriae
E. lata
F. fujikuroi
F. graminearum
F. oxysporum
F. solani
F. verticillioides
M. phaseolina
N. parvum
P. aleophilum
P. digitatum
P. teres
P. tritici-repentis
S. nodorum
S. sclerotiorum
V. albo-atrum
V. dahliae
Conserved proteins
BLASTP NR hits
Species-specific proteins

Wood-decaying necrotroph

Basidiomycota

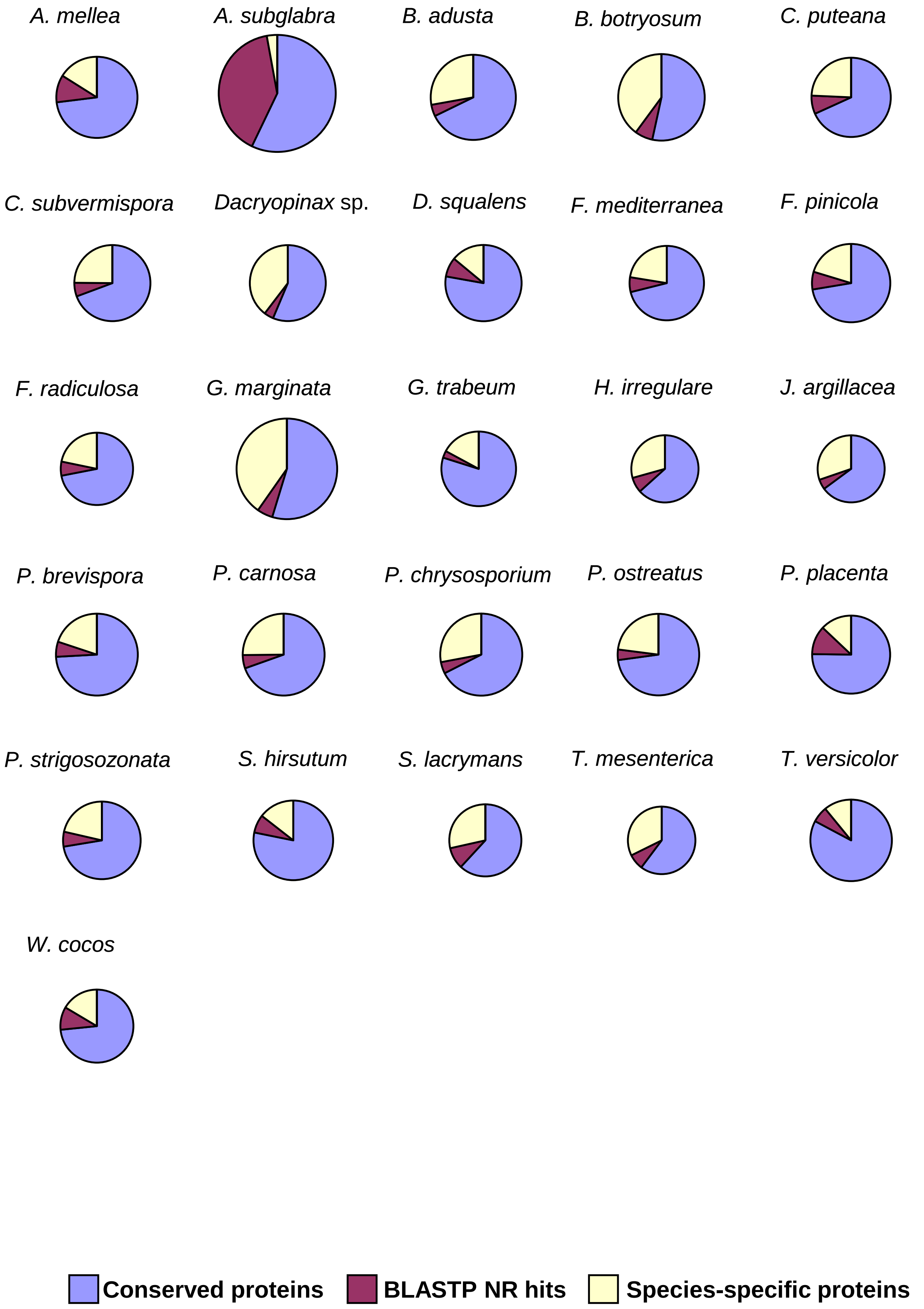

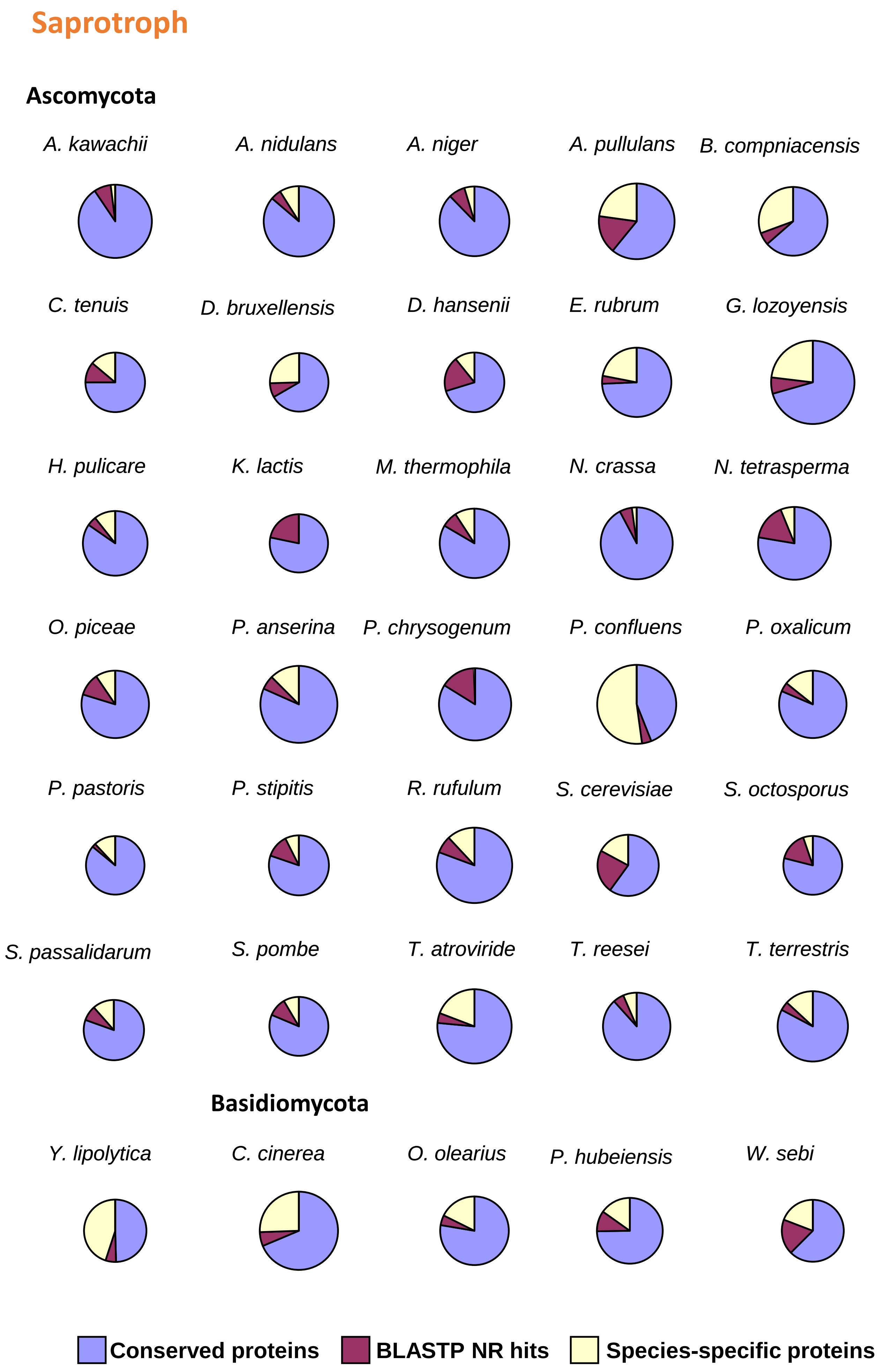
Saprotroph
Ascomycota
A. kawachii
A. nidulans
A. niger
A. pullulans
B. compniacensis
C. tenuis
D. bruxellensis
D. hansenii
E. rubrum
G. lozoyensis
H. pulicare
K. lactis
M. thermophila
N. crassa
N. tetrasperma
O. piceae
P. anserina
P. chrysogenum
P. confluens
P. oxalicum
P. pastoris
P. stipitis
R. rufulum
S. cerevisiae
S. octosporus
S. passalidarum
S. pombe
T. atroviride
T. reesei
T. terrestris
Basidiomycota
Y. lipolytica
C. cinerea
O. olearius
P. hubeiensis
W. sebi
Conserved proteins
BLASTP NR hits
Species-specific proteins

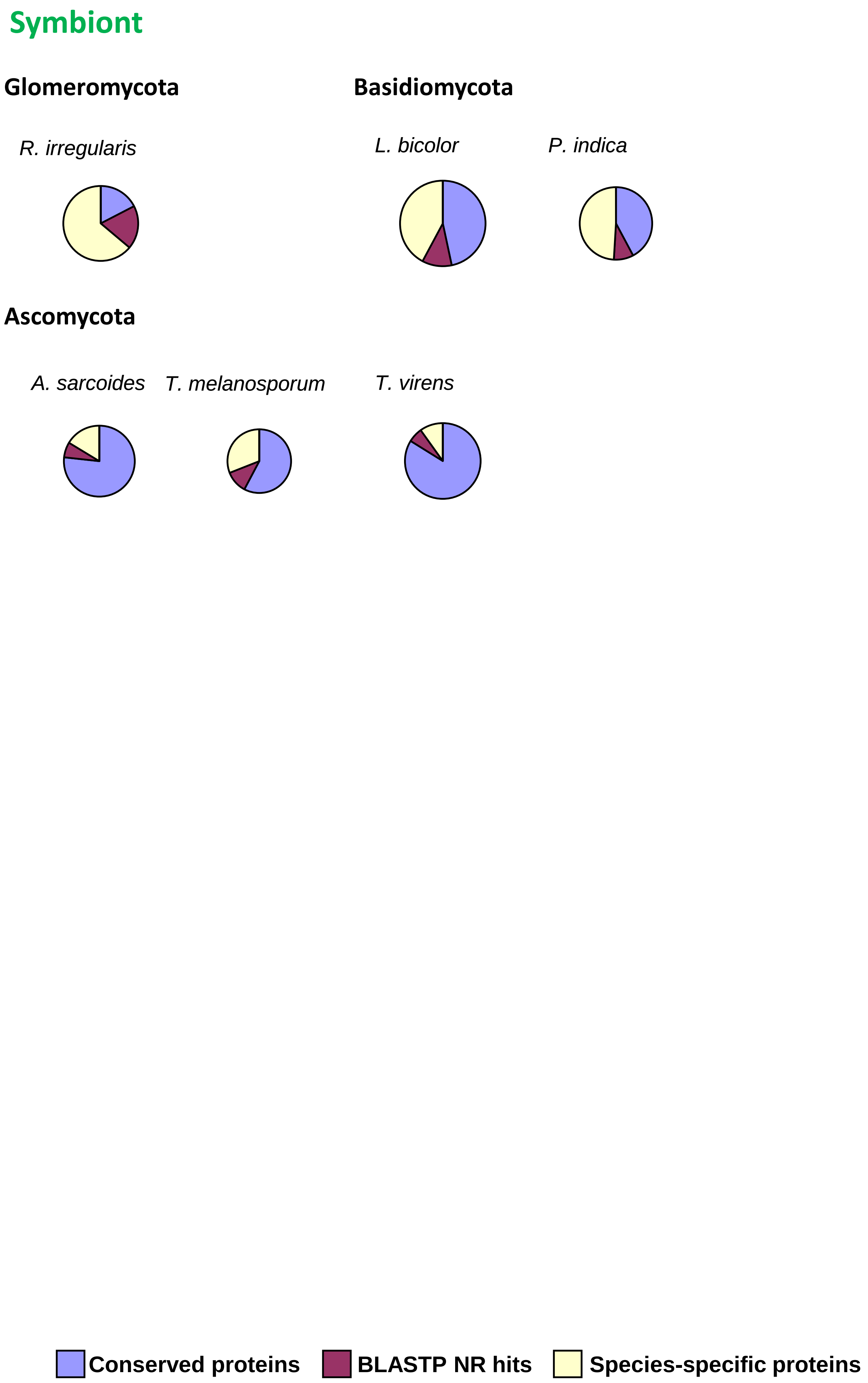
Symbiont
Glomeromycota
R. irregularis
Basidiomycota
L. bicolor
P. indica
Ascomycota
A. sarcoides
T. melanosporum
T. virens
Conserved proteins
BLASTP NR hits
Species-specific proteins
