# Supplementary material for: Kingdom-Wide Analysis of Fungal Small Secreted Proteins (SSPs) Reveals their Potential Role in Host Association
Source: Front Plant Sci. 2016 Feb 19;7:186. doi: 10.3389/fpls.2016.00186 (PMC4759460; doi:10.3389/fpls.2016.00186)

**Figure S5 | Relationship between the total proteome size and the refined secretome including its contents.**

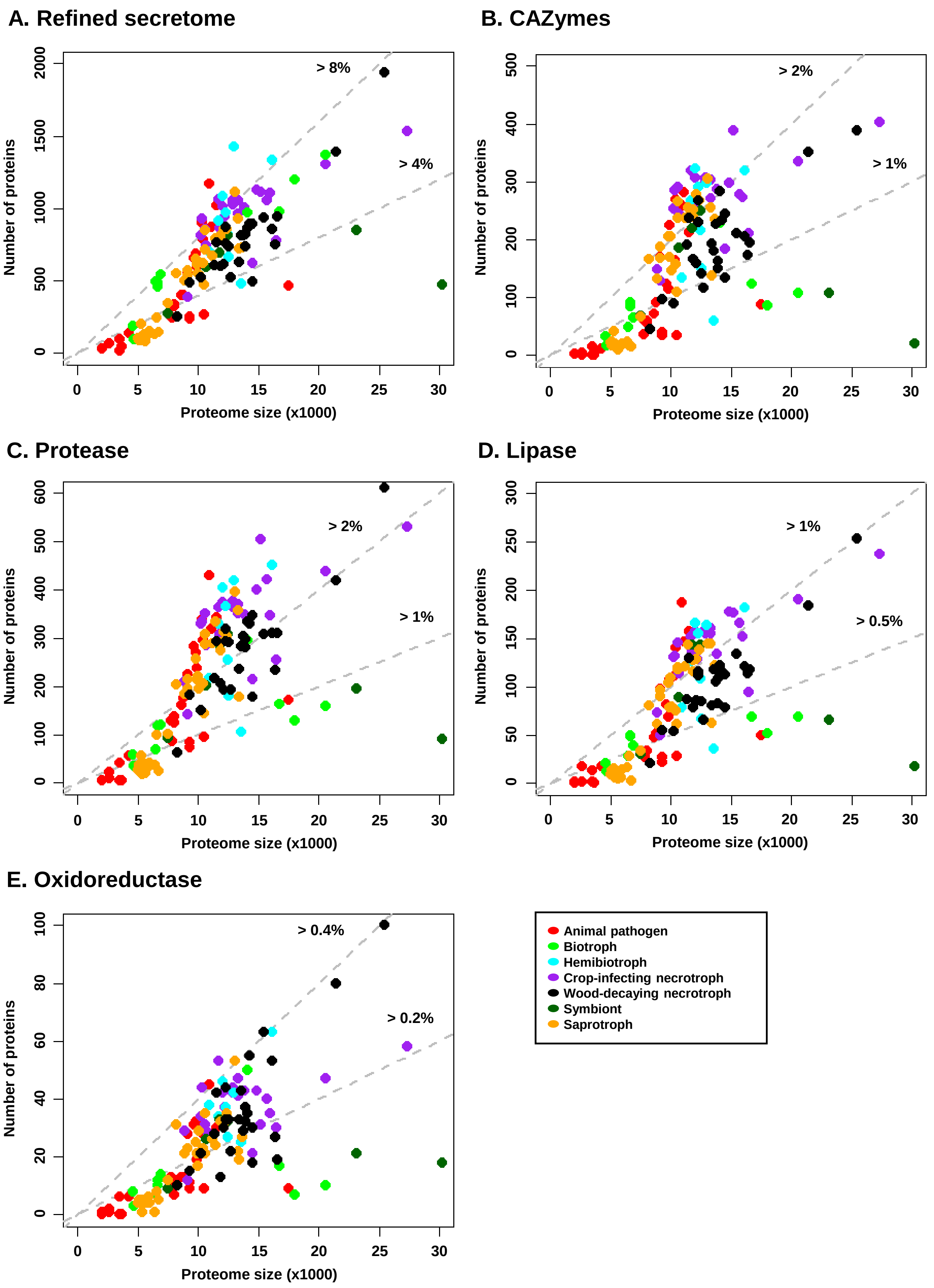

Supplement: Supplementary file 9 [file Presentation5.PDF]
