# Supplementary material for: Kingdom-Wide Analysis of Fungal Small Secreted Proteins (SSPs) Reveals their Potential Role in Host Association
Source: Front Plant Sci. 2016 Feb 19;7:186. doi: 10.3389/fpls.2016.00186 (PMC4759460; doi:10.3389/fpls.2016.00186)
Supplement: Supplementary file 10 [file Presentation6.PDF]

Figure S6 | Evolution of selected conserved small secreted protein families.

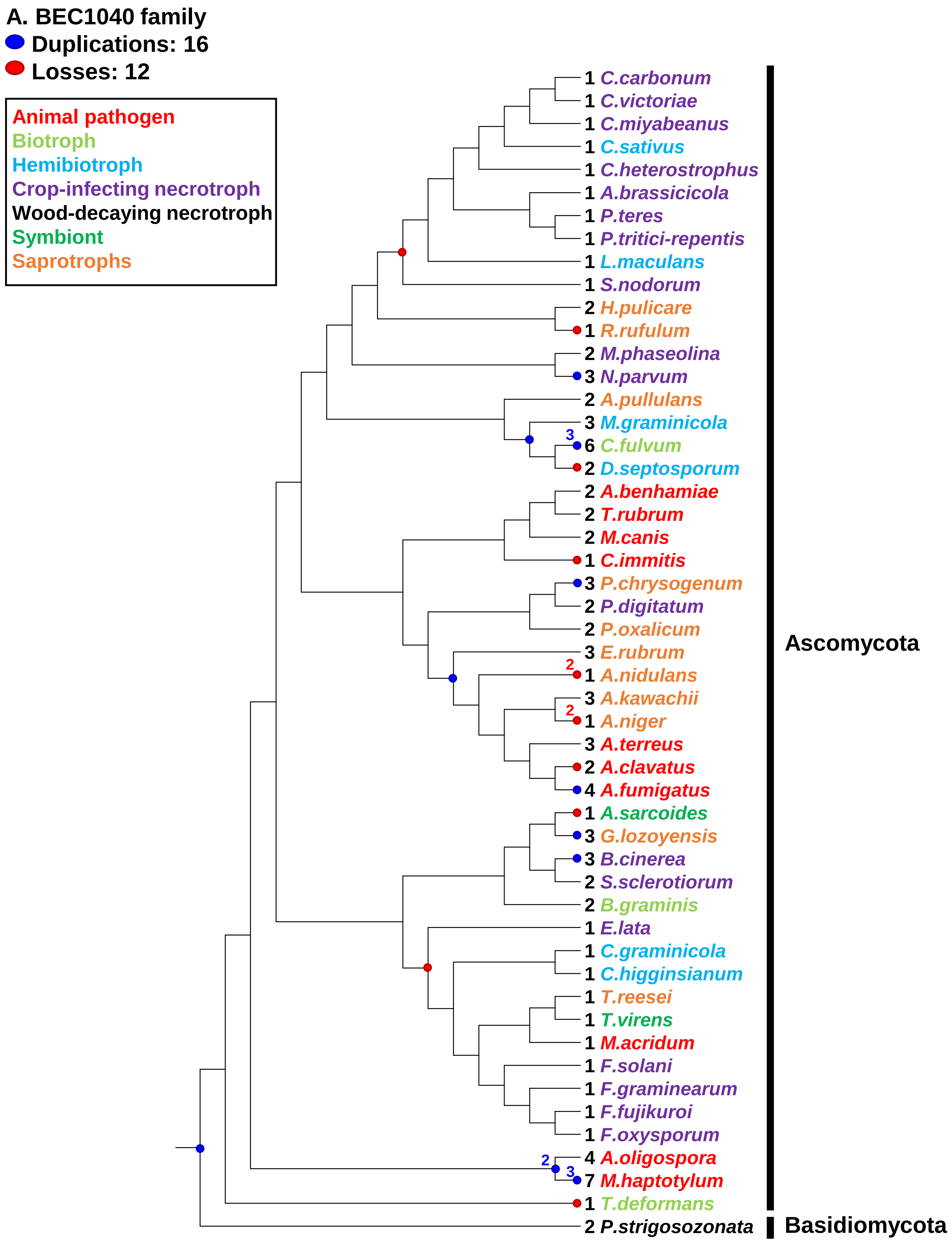

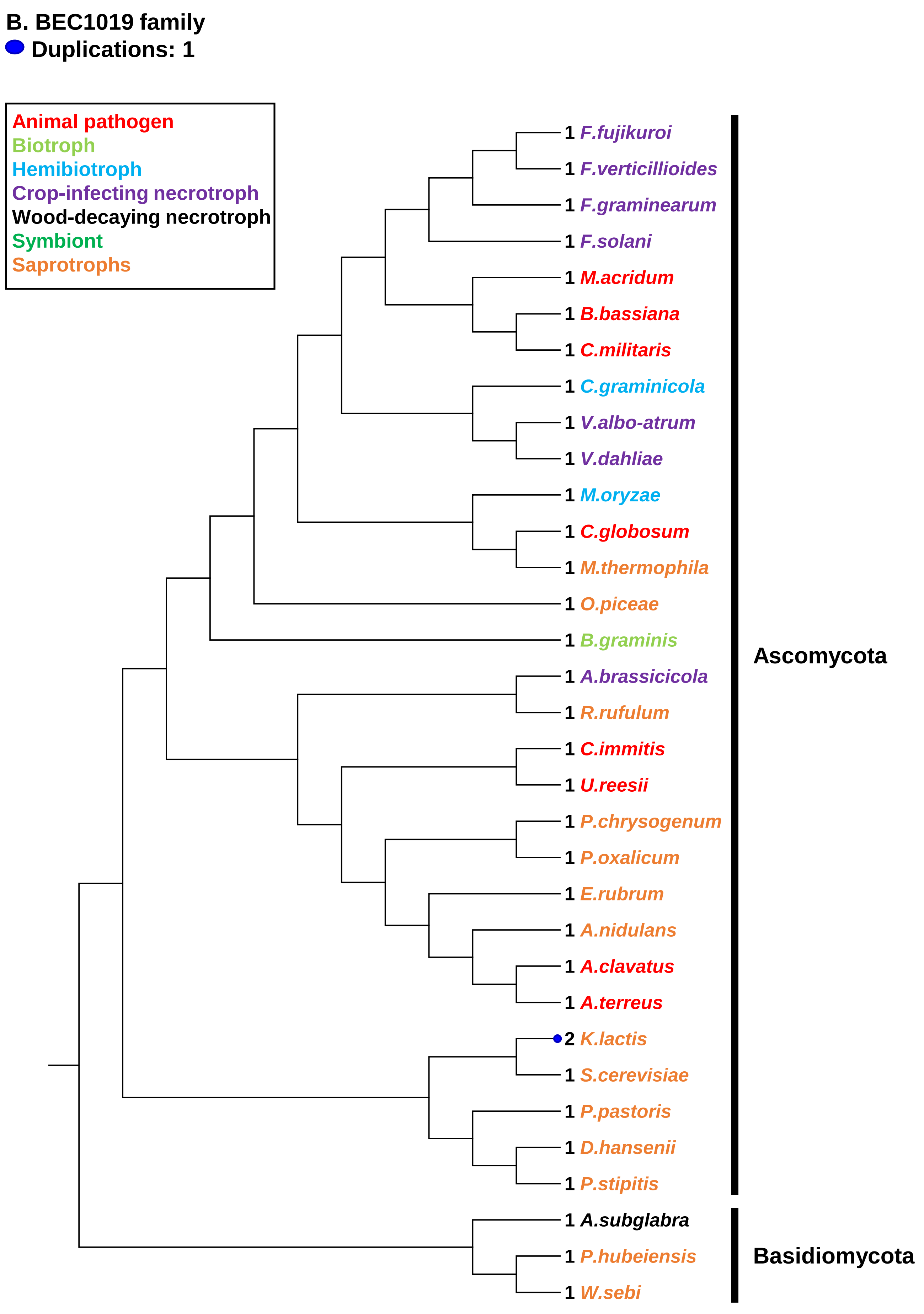
B. BEC1019 family
Duplications: 1
Animal pathogen
Biotroph
Hemibiotroph
Crop-infecting necrotroph
Wood-decaying necrotroph
Symbiont
Saprotrophs
1 F.fujikuroi
1 F.verticillioides
1 F.graminearum
1 F.solani
1 M.acridum
1 B.bassiana
1 C.militaris
1 C.graminicola
1 V.albo-atrum
1 V.dahliae
1 M.oryzae
1 C.globosum
1 M.thermophila
1 O.piceae
1 B.graminis
1 A.brassicicola
1 R.rufulum
1 C.immitis
1 U.reesii
1 P.chrysogenum
1 P.oxalicum
1 E.rubrum
1 A.nidulans
1 A.clavatus
1 A.terreus
2 K.lactis
1 S.cerevisiae
1 P.pastoris
1 D.hansenii
1 P.stipitis
1 A.subglabra
1 P.hubeiensis
1 W.sebi
Ascomycota
Basidiomycota

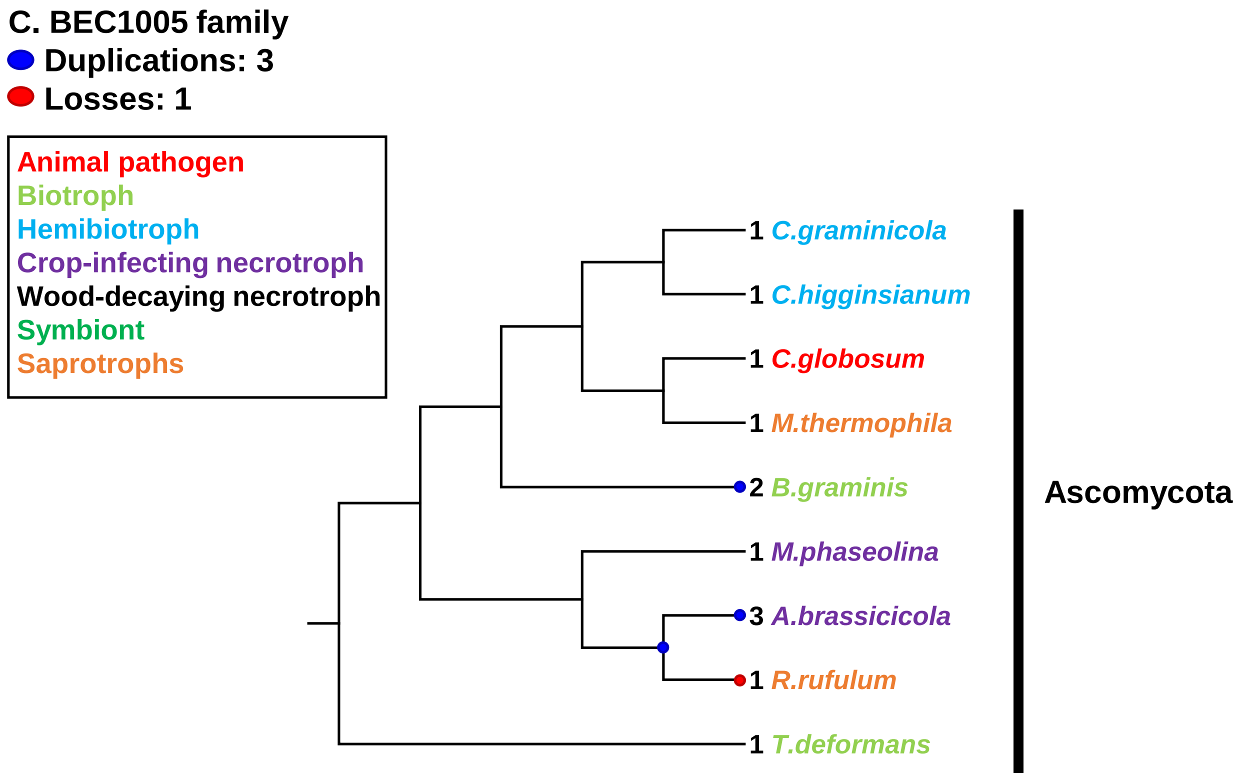

C. BEC1005 family
Duplications: 3
Losses: 1
Animal pathogen
Biotroph
Hemibiotroph
Crop-infecting necrotroph
Wood-decaying necrotroph
Symbiont
Saprotrophs
1 C.graminicola
1 C.higginsianum
1 C.globosum
1 M.thermophila
2 B.graminis
1 M.phaseolina
3 A.brassicicola
1 R.rufulum
1 T.deformans
Ascomycota
