# Supplementary material for: Kingdom-Wide Analysis of Fungal Small Secreted Proteins (SSPs) Reveals their Potential Role in Host Association
Source: Front Plant Sci. 2016 Feb 19;7:186. doi: 10.3389/fpls.2016.00186 (PMC4759460; doi:10.3389/fpls.2016.00186)
Supplement: Supplementary file 12 [file DataSheet2.DOCX]

***Supplementary Material***

**Kingdom-wide analysis of fungal small secreted proteins (SSPs) reveals their potential role in host association**

**Ki-Tae Kim^1,2^, Jongbum Jeon^1,3^, Jaeyoung Choi^1,3#^, Kyeongchae Cheong^1,3^, Hyeunjeong Song^1,3^, Gobong Choi^1,3^, Seogchan Kang^4^ and Yong-Hwan Lee^1,2,3,5^***

^1^Fungal Bioinformatics Laboratory, Seoul National University, Seoul 151-921, Korea

^2^Department of Agricultural Biotechnology, Seoul National University, Seoul 151-921, Korea

^3^Interdiscplinary Program in Agricultural Genomics, Seoul National University, Seoul 151-921, Korea

^4^Department of Plant Pathology & Environmental Microbiology, The Pennsylvania State University, University Park, PA 16802, USA

^5^Center for Fungal Genetic Resources, Center for Fungal Pathogenesis, Plant Genomics and Breeding Institute, Research Institute of Agriculture and Life Sciences, Seoul National University, Seoul 151-921, Korea

*** Correspondence:** Prof. Yong-Hwan Lee, Fungal Bioinformatics Laboratory, Seoul National University, Seoul 151-921, Korea

[yonglee@snu.ac.kr](mailto:yonglee@snu.ac.kr)

^#^ Current address: The Samuel Roberts Noble Foundation, Ardmore, OK 73401, USA

1. **Supplementary Figures**

**Figure S1 | Comparison between the refined secretomes and the FSD secretomes of individual species.**

The FSD secretomes include all possible secretory proteins archived in FSD. Class SP is secretory proteins which were predicted only by SignalP 3.0 and TMHMM. Refined secretomes are color-coded by lifestyles and the species are arranged in increasing size of the refined secretomes within the lifestyles.

**Figure S2 | Phylogenetic tree for 136 species and their refined secretome.**

The phylogenetic relationship of 136 fungal species are shown as a tree, which were constructed using CVTree with whole proteomes. The lifestyles of each species are color-coded and their predicted size of refined secretomes are shown as bar graphs.

**Figure S3 | Protein length distributions within the proteins of other functions.**

The proportion of protein length for individual species are shown as pie graphs. The size of pie graphs represent the amount of proteins of other functions.

**Figure S4 | Species-specific proteins in the proteins of other functions.**

The proportion of species-specific proteins for individual species are shown as pie graphs. The size of pie graphs represent the amount of proteins of other functions.

**Figure S5 | Relationship between the total proteome size and the refined secretome including its contents.**

(A) The relationship between the size of refined secretomes and the predicted proteomes. (B) The size of CAZymes against the predicted proteomes. (C) The size of proteases against the predicted proteomes. (D) The size of lipases against the predicted proteomes. (E) The size of oxidoreductases against the predicted proteomes.

**Figure S6 | Evolution of selected conserved small secreted protein families.**

Gene duplication events are represented as blue nodes and losses as red nodes. The lifestyles of individual species are color-coded. The CSSP families include (A) BEC1040, (B) BEC1019 and (C) BEC1005.

1. **Supplementary Tables**

**Table S1 | List of 136 species analyzed, their lifestyle, phylogeny, secretome and its content, number of SSSPs and genome reference**

**Table S2 | Summary of SSSPs InterPro term annotation**

**Table S3 | List of 49 PHI-base effectors used for mapping onto SSSPs and clustering with CSSPs**

**Table S4 | Summary for genomic contexts of SSSPs encoding genes**

1. **Supplementary Data**

**Data S1 | SSSP sequences for 133 species in fasta format**
